# A hybrid flower pollination algorithm–deep learning approach for strength prediction of sustainable recycled fine aggregate concrete with GUI-based implementation

**DOI:** 10.1038/s41598-026-51308-1

**Published:** 2026-05-01

**Authors:** Kumar Shubham, M. K. Diptikanta Rout, Bibhu Prasad Mishra, Sabyasachi Biswas, Gobinath Ravindran

**Affiliations:** 1 Centre for Promotion of Research, Graphic Era (Deemed to be University), Dehradun, 248001 Uttarakhand India; 2 Department of Civil Engineering, Indian Institute of Technology Guwahati, Guwahati, 738039 Assam India; 3 School of Infrastructure & Planning, Odisha University of Technology & Research, Bhubaneswar, 751029 Odisha India; 4 Department of Civil Engineering, National Institute of Technology Durgapur, Durgapur, 713209 West Bengal India; 5 Symbiosis Centre for Research and Innovation, Symbiosis International (Deemed University), Pune, 412155 Maharashtra India

**Keywords:** WRFA, Mineral Admixtures, Flower Pollination Algorithm (FPA), Sensitivity Analysis, Engineering, Materials science, Mathematics and computing

## Abstract

This study presents an integrated experimental–machine learning framework for evaluating and predicting the compressive strength of sustainable rigid pavement concrete incorporating 50% washed recycled fine aggregates (WRFA) as a replacement for natural fine aggregates, together with zirconia silica fume (ZSF) and steel slag (SS) as supplementary cementitious materials. The novelty lies in the development of a hybrid Flower Pollination Algorithm–optimized deep neural network (FPA–DNN) that enhances prediction accuracy, robustness to noise, and interpretability through SHAP-based analysis, alongside a GUI-enabled decision-support tool for real-time application. Experimental findings indicated that 50% WRFA reduced compressive strength by 29.6%, 25.2%, and 23.9% at 7, 28, and 90 days, respectively, relative to the control mix. Among the investigated formulations, 20% SS cement replacement (SS20) provided the most favorable performance, limiting flexural strength reduction to approximately 3% and split tensile strength loss to 3.8% at 28 days while maintaining pavement-grade requirements. SEM-based microstructural evaluation of the optimal WRFA50 + SS20 mixture confirmed a denser cementitious matrix, reduced porosity, and improved interfacial transition zone bonding, attributed to SS-induced secondary hydration. A dataset comprising 264 samples (103 laboratory-generated and 161 collected from screened literature) was used to train and compare five regression models: kNN, RF, ANN, DNN, and FPA-DNN. Using 10-fold cross-validation, the FPA-DNN achieved the highest predictive accuracy, yielding testing performance of R² = 0.96 ± 0.006, RMSE = 2.87 ± 0.09 MPa, and MAPE = 4.38 ± 0.13%, outperforming kNN (R² = 0.85), RF (0.86), ANN (0.89), and standalone DNN (0.91). A noise-robustness assessment, conducted by applying additive white Gaussian noise to the target variable (σ up to ~ 1.6% of mean compressive strength), further confirmed the stability of the proposed model, with FPA-DNN retaining R² > 0.90 at the highest noise level (*p* = 0.20), whereas conventional models exhibited greater degradation. SHapley Additive ExPlanations (SHAP)-based interpretability identified cement content and steel slag as the most influential positive predictors, while WRFA showed a replacement-dependent effect. The proposed framework offers an interpretable and noise-resilient approach for strength prediction of recycled aggregate concrete; however, the present study is limited to a fixed WRFA replacement level and compressive-strength-focused modelling. Future work should extend validation to broader WRFA sources, wider replacement ranges, and durability-based performance indicators for long-term pavement applications.

## Introduction

Concrete is one of the essential construction materials that is widely used in civil infrastructure development^1^. In the transportation sector, the rigid pavement is a significant application of concrete due to high durability than asphalt pavement, less maintenance-intensive and longer service life^2^. India has the second-largest road network in the world, with a total of 67.73 lakh roads, according to a report from the Ministry of Road Transport and Highways (MORTH)^3^. The rapid expansion of the road network puts thrust on usage of resources and there is a need to preserve the natural aggregates for future generations in the sense of resource conservation^4^. For balancing the economy and enhancing sustainable growth, the use of recycled asphalt aggregates (RAP) proved to be a better alternative to conventional construction materials in concrete pavements^5^. RAP is a material created by grinding up old asphalt pavement and reusing it as an aggregate in new asphalt pavement^6^. However, RAP can also be used as an aggregate in concrete pavement for sustainable growth in the construction industry^7^. RAP in the concrete pavement can help reduce construction costs, since it is often less expensive than using virgin aggregates^8,9^. In addition, using RAP in concrete pavement may require changes to the mix design, as RAP aggregates may have different properties than natural aggregates^10,11^. Washed Recycled Fine Aggregates (WRFA) refers to a recycled material that is finely crushed and screened, obtained through the recycling process of asphalt pavement. This process involves taking old asphalt pavement materials and transforming it into finely crushed and screened particles^12^. The fine aggregates typically have a particle size smaller than 4.75 mm (No. 4 sieve)^13^. The process of WRFA typically involves collection and stockpiling, screening, washing, drying, and quality control^14,15^.

The existing research reported that the usage of 50% WRFA enhanced the strength of RAP concrete mixes in cement concrete pavements without affecting any properties of concrete, while using 100% RAP as a replacement degraded the strength due to the asphalt coating layer around the aggregates^16–18,18^. However, use of industrial by-products, such as silica fume (SF), fly ash (FA), bagasse ash (BG), Rice husk ash (RHA), ground granulated blast-furnace slag (GGBFS), and other supplementary cementitious materials (SCMs) in RAP concrete mixtures, in order to increase the strength of RAP and reduces the cement content which is the priority for sustainable growth of construction industry^19,21,22,22^. Brand et al.^20^ evaluated that the reduction of strength in cement concrete pavements containing fine RAP aggregates was less, while^23^ found that replacing up to 50% of fine RAP aggregates might result in a benchmark strength, i.e. 40 MPa, which was a beneficial finding for the construction of concrete pavements. According to^24^, approximately 40–50% strength of concrete mix could be enhanced with the addition of steel fibers incorporating with fine RAP aggregates in rigid pavement, whereas Gupta et al.^25^ found that the addition up to 15% of jarosite can improve the strength properties of concrete pavements for low-volume roads. Additionally, Debbrama et al.^26^ examined the performance of roller compacted concrete pavement (RCCP) mixes that included 50% dust-contaminated RAP sourced from different agricultural and industrial sources. It was further observed that incorporating such materials enhanced the durability and mechanical properties of the concrete mixes. In addition to this, Rezai et al.^27^ correlated the better relationship between the fine RAP content and the compressive strength of concrete. Debbarma et al.^28^ found that compressive, split tensile, and flexural strengths significantly increase with the addition of cementitious admixtures to RAP, leading to a decrease in the cement content. Rout et al.^29^ confirmed that WRFA have achieved the benchmark of strength properties when used within the 50% substitution level in base courses. Owing to these findings, existing literature recommended the use of WRFA in pavement base layers to satisfy the required strength criteria. Furthermore, the utilization of these aggregates enhances resource efficiency by reducing the consumption for natural fine aggregates and promoting the reuse of RAP materials.

### Application of machine learning in strength prediction

Recently, many studies have also focused on the understanding about applications of Machine learning (ML) and deep learning (DL) in the civil engineering^30,32,33,33^. ML can be used in civil engineering to enhance the effectiveness, safety, and sustainability of infrastructure and construction projects, hence lowering costs and improving performance^34,35^. Several studies have been employed on ANN for predicting the strength, such as compressive, tensile, flexural, shear, on different types of concrete^36,38,39,39^. Debbarma and Ransinchung^40^ attempted to predict the compressive strength of RCCP incorporating RAP aggregates using ANN algorithms with the Bayesian Regularization (BR) training function. The proposed model has shown greater accuracy (R^2^ = 0.994) and high predictive performance using a 13-9-9-1 multilayer ANN architecture. Similarly, Singh et al.^41^ reported that the BR-ANN model has high accuracy, i.e. R^2^ value of 0.9973 with a corresponding lower error of 0.0305 for forecasting the unconfined compressive strength of cement-treated base (CTB) mixes incorporating RAP aggregates. The validation performance of the considered model was 0.0065 MPa at 36 epochs. Getahun et al.^42^predicted the compressive and tensile strength of concrete using limited experimental data samples by a three-layer perception ANN model incorporating RAP as alternative aggregate along with RHA as agricultural waste. The research found that the predictive results of the neural network were deviated by 2.088 and 2.095% with corresponding CS and TS, respectively. Moreover, Duan at al^43^. demonstrated that the implementation of ANN exhibited as a superior tool for predicting the compressive strength of recycled concrete aggregate (RCA) compared to virgin aggregates. The metric scores (R^2^, MSE and MAPE) have shown very little relative error by constructing a 14–16-1 hidden layer of neurons and the resulting model is a good fit for the training dataset. Ullah and Zainab^34^ concluded that the ANN model is efficient and reliable for predicting the permanent deformation of RAP aggregates used as base course material in asphalt pavements. The ANN architecture model yielded highly accurate results, closely aligning with the experimentally measured values. This was evident through achieving the lowest Mean Squared Error (MSE) value at the 70th epoch. Lam et al.^44^ developed various models based on an ANN model to predict the compressive strength of RCCP containing mineral admixture. The observed values showed a satisfactory prediction in the case of the Fuzzy Logic (FL) model, as it contains fuzzy rules with R² values of 0.96 for the training dataset and 0.835 for the testing dataset. To further improve the ANN model, several studies have attempted to develop a DNN-based model for more accurate prediction of concrete strength. Thus, it is evident that usage of ML methods to predict the strength characteristics can be effectively employed in civil engineering purpose.

The objective of the present study aims to investigate the performance and predictability of sustainable concrete incorporating WRFA and industrial by-products. Specifically, the study evaluates the feasibility of using WRFA as a partial replacement for natural fine aggregates in M40 grade concrete for rigid pavement applications and examines the combined influence of zirconia silica fume (ZSF) and steel slag (SS) on mechanical properties to identify an optimal mix proportion. In parallel, multiple ML models including k-nearest neighbors (kNN), random forest (RF), artificial neural networks (ANN), and deep neural networks (DNN) were developed and compared for compressive strength prediction. A hybrid Flower Pollination Algorithm–based deep neural network (FPA–DNN) model was further proposed to enhance predictive accuracy and generalization. Explainable artificial intelligence (XAI) tools such as SHAP, partial dependence plots (PDP), and individual conditional expectation (ICE) plots were employed to interpret the model predictions and quantify the influence of input parameters on compressive strength. Furthermore, the effect of Additive White Gaussian Noise (AWGN) on model performance is investigated to assess the robustness and stability of the proposed models under data uncertainty and noisy input conditions. Finally, a graphical user interface (GUI) was developed to facilitate real-time and user-friendly strength prediction for practical engineering applications.

## Novelty of the present study

The novelty of this study lies in presenting an integrated experimental and ML framework specifically developed for WRFA-based rigid pavement concrete incorporating industrial by-products (steel slag and zirconia silica fume). While previous studies have reported ML-based strength prediction for sustainable concretes, the present work is distinct due to its explicit focus on WRFA as the primary sustainability component for pavement-grade concrete, supported by a combined dataset from laboratory experiments and screened literature. The study further investigates the combined role of SS and ZSF additives, including the identification of an optimum SS dosage for strength enhancement. To overcome the overfitting and generalization limitations commonly associated with standalone deep neural networks trained on limited and heterogeneous datasets, an optimized hybrid FPA-DNN model was proposed in this research. Model transparency is enhanced through SHAP-based interpretability analysis, which quantifies the influence of each mix-design parameter on compressive strength prediction. In addition, the robustness of the developed models is examined through a white Gaussian noise simulation to evaluate predictive stability under noisy input conditions. Finally, an ML-based GUI is developed to facilitate real-time strength prediction and practical implementation, thereby bridging the gap between laboratory research and field-level application of sustainable pavement concrete technologies.

## Experimental investigation

### Materials

**Fig. 1:**
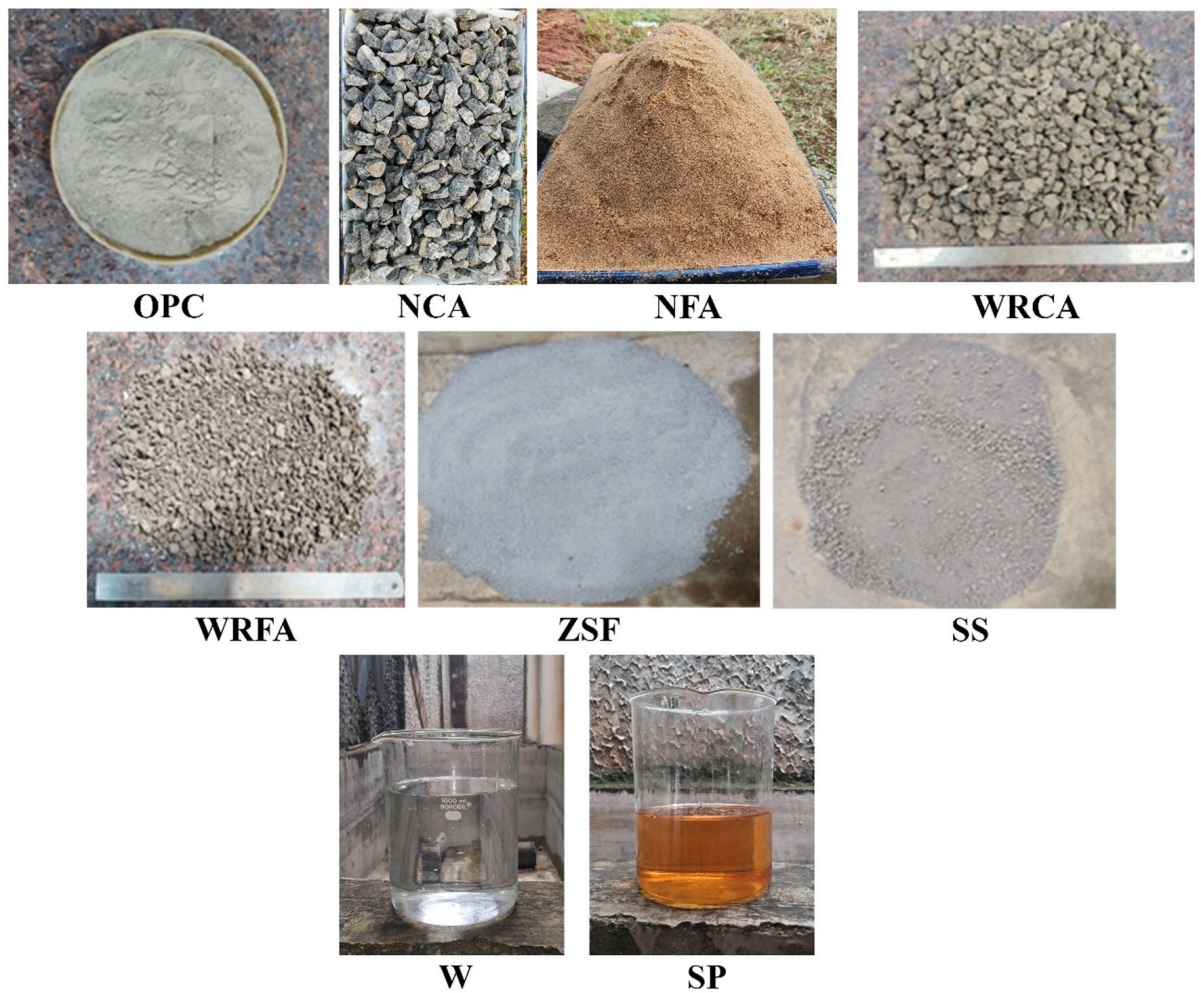
Constituent materials used in the preparation of concrete mixes.

In this present research, various materials, including natural aggregate (NA), washed recycled coarse aggregates (WRCA), WRFA, ZSF and SS were utilized to prepare concrete mix. The visual appearance of all the materials considered is referred to in Fig. 1. Ordinary Portland Cement (OPC) of 53 grade was utilized, which confirms to  : IS 12269:2013 standards ^45^. The cement had a specific gravity of 3.15, a standard consistency of approximately 31%, and an initial and final setting time of about 35 min and 520 min, respectively. The fineness was determined using the Blaine air permeability method and found approximately around 320 m²/kg. The chemical composition of all the binder materials determined using X-ray Fluorescence (XRF) analysis and is presented in Table 1.

**Table 1:** Chemical composition of OPC, ZSF and SS.

Composition	SiO_2_	CaO	Fe_2_O_3_	MgO	Al_2_O_3_	MnO	KO	Na_2_O	TiO_2_	LIO
OPC (%)	37.28	48.76	2.75	2.14	5.8	0.02	0.72	0.82	0.37	1.34
ZSF (%)	92.85	0.45	0.94	0.36	1.08	0.05	0.75	1.6	0.15	1.77
SS (%)	14.34	48.62	28.34	1.88	1.46	0.39	0.52	0.67	0.92	2.86

**Fig. 2:**
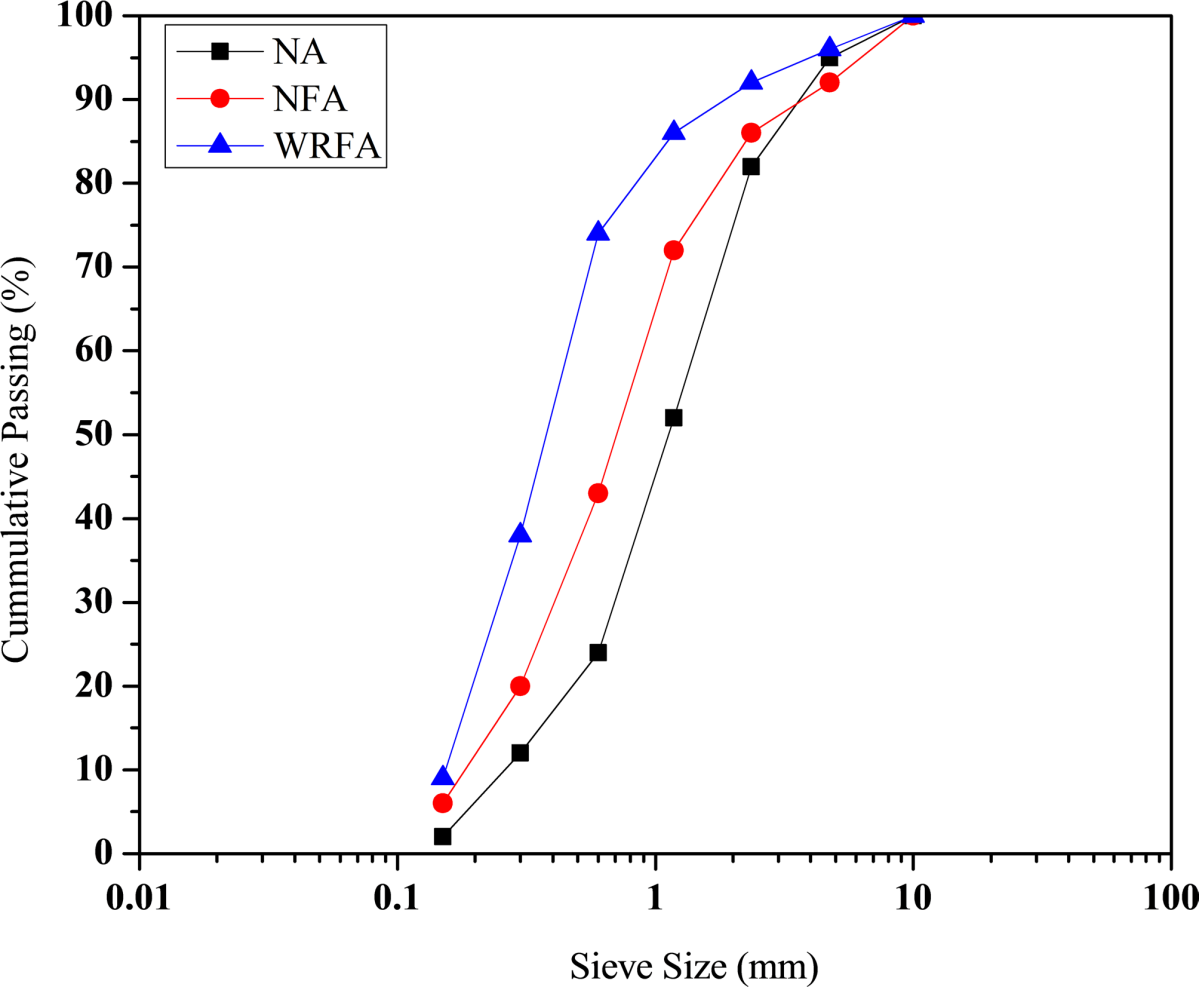
Particle Size Distribution Curve of NA, NFA and WRFA.

RAP was acquired from various local sites in the state of Jharkhand, where the bituminous pavement is designed for 20 years. The WRFA used in this study were obtained from RAP sourced from a local asphalt recycling plant. Prior to use, the RAP fines were subjected to a controlled washing protocol to remove loose dust, residual asphalt coatings, and deleterious materials. The washing process was carried out using a liquid solvent (petroleum ether), selected for its effectiveness in dissolving residual bitumen without altering the mineral composition of the aggregates. The RAP fines were immersed in the solvent and mechanically agitated for 30 min, followed by decantation of the solvent containing dissolved asphalt residues. This procedure was repeated for three successive cycles to ensure effective removal of residual coatings. After washing, the aggregates were thoroughly rinsed with clean water to eliminate any solvent traces and subsequently air-dried at ambient laboratory conditions (25 ± 2 °C) until a constant mass was achieved. Residual bitumen content was evaluated using solvent extraction methods and was found to be less than 0.5%. The fines index of WRFA ranged between 4 and 6%, confirming compliance with fine aggregate quality requirements. Particle-size distribution analysis showed that the WRFA particles were within the size range of 0.075–4.75 mm. The grain size distribution curve of all the considered aggregates is presented in Fig. 2. The ZSF admixture used in this study was produced from zirconia-based compounds through a controlled fusion process, resulting in ultrafine particles with sizes smaller than 1 μm (0.001 mm). In addition, SS employed in the present investigation was sourced from the Tata Steel Plant, Jamshedpur, India.

ZSF and SS were selected due to their regional availability and complementary cementitious roles. ZSF is an ultrafine silica-rich material that enhances micro-filling and pozzolanic reaction, whereas SS contributes CaO-rich hydraulic reactivity and improves strength development. While fly ash and GGBS are extensively studied, ZSF has limited exploration in pavement-grade concrete; therefore, the present study emphasizes locally available industrial wastes and their combined role in WRFA-based sustainable concrete.

### Experimental design

The mix proportions of the considered RAP mixes are tabulated in Table 2. The specifications outlined in IRC: 44–2017 adhered to for the mix design of cement concrete pavements as per the guidelines set by the Indian Road Congress (IRC)^46^. The water to cement (W/C) ratio considered for the mix design is 0.46. The study involves casting and testing M40 concrete mixes with different replacements of natural fine aggregates (NFA) with WRFA in accordance with Indian Standard (IS) 383–1970 guidelines^47^. In the present investigation, 50% of WRA are utilized as partial replacements for NFA for the preparing the concrete mix. The study is examining partial substitutions of cement content by 10%, 20%, and 30% of both ZSF and SS admixtures for the concrete mixes. A sulfonated naphthalene-based superplasticizer (SP) was used in all mixes to maintain the W/C ratio.

**Table 2:** Mix proportions of the considered concrete mixes in kg/m^3^.

Mix Designation	OPC	NCA	NFA	WRFA	ZSF	SS	Water	SP
Control Mix	460	1430	685	0	0	0	185	2.30
WRFA50	460	715	342.5	342.5	0	0	185	2.76
ZSF5	437	715	342.5	342.5	23	0	185	2.84
ZSF10	414	715	342.5	342.5	46	0	185	2.90
ZSF15	391	715	342.5	342.5	69	0	185	2.93
SS10	414	715	342.5	342.5	0	46	185	3.31
SS20	368	715	342.5	342.5	0	92	185	3.31
SS30	322	715	342.5	342.5	0	138	185	3.06
ZSF10 + SS20	322	715	342.5	342.5	46	92	185	3.22
ZSF10 + SS30	276	715	342.5	342.5	46	138	185	3.04

## Data acquisition for modeling and statistics

For performing the ML based modeling the dataset used in this study contains nine input parameters (C, NCA, NFA, WRCA, WRFA, ZSF, SS, W, SP) as the predictors (input variable) with one target attribute (output variable) as compressive strength (f_c_). The dataset used in this study comprised a total of 264 data samples were gathered from the from laboratory experiments as well as relevant literatures. From the entire datasets, 103 samples generated from laboratory experiments conducted in the present work which were used for the development and validation of the ML models. Additionally, 161 data samples were extracted from relevant peer-reviewed literature to support the development of the ML models^48,50–57,55^. This strategy was adopted to incorporate variability associated with WRFA source, RAP aging, aggregate mineralogy, and regional production conditions. Therefore, the developed ML models are not restricted to a single material batch or a specific geographical region. Nevertheless, it is acknowledged that WRFA characteristics may vary significantly depending on RAP origin, residual binder content, and processing methods. Hence, model reliability is expected to be strongest within the range of input parameters represented in the compiled dataset.

The literature data were carefully screened to ensure compatibility with the experimental dataset in terms of material composition, curing conditions, testing age, and reported f_c_. The dataset was divided into 70% for training and 30% for testing, with the training portion further subjected to internal validation to ensure model stability and prevent overfitting. A comprehensive statistical summary of all variables, including their minimum, maximum, mean, and standard deviation values is presented in Table 3. Since the dataset was compiled from both laboratory experiments and peer-reviewed literature, careful screening was performed during data acquisition to minimize unrealistic extreme observations. Data points were retained only when they were consistent with comparable material composition, curing/testing conditions, and reported strength ranges, thereby reducing the possibility of anomalous outliers affecting model training.

**Table 3:** Statistical description of the dataset.

Input Variables	Notation	Role	Mean	Standard Deviation	Minimum	Median (50%)	Maximum
Cement (kg/m^3^)	C	Input	396.71	53.24	270	411	464
Natural Coarse Aggregates (kg/m^3^)	NCA	Input	888.758	313.93	0	723	1438
Natural Fine Aggregates (kg/m^3^)	NFA	Input	450.382	301.91	0	338	1149
Washed Recycled Coarse Aggregates (kg/m^3^)	WRCA	Input	490.965	346.21	0	0	1360
Washed Recycled Fine Aggregates (kg/m^3^)	WRFA	Input	295.258	238.17	0	47.75	1078
Zirconia Silica Fume (kg/m^3^)	ZSF	Input	25.896	46.187	0	0	230
Steel Slag (kg/m^3^)	SS	Input	50.241	50.242	0	0	145
Water (kg/m^3^)	W	Input	161.913	47.477	0	16.50	198
Super-plasticizer (kg/m^3^)	SP	Input	3.24	2.86	0	2.70	16.0
Compressive strength (MPa)	f_c_	Output	41.487	3.29	31.08	39.70	49

### Data normalization

Prior to model training, the dataset was normalized to ensure consistent feature scaling and effective convergence of the ML algorithms for avoiding dominance of high-magnitude features. The collected entire data-points consist of variables measured in different units and ranges^56^. In this study, all input parameters were normalised to a uniform scale using a min–max normalization approach, which transforms each variable into a standardised range between 0 and 1. This ensures that every input variable contributes equally during model training, improving convergence and predictive stability. All input features were scaled to a consistent range using Min–Max normalization technique as expressed in Eq. (1)

1 $$\:{K}_{\mathrm{Norm}}=\frac{K\:-\:{K}_{\mathrm{Min}}}{{K}_{\mathrm{Max}\text{}}-{\:K}_{\mathrm{Min}}}$$

In this equation, K is the original input value, K_Min_ and K_Max_ are the minimum and maximum values of the corresponding input variable, and $\:{K}_{\mathrm{Norm}}\:$is the normalized value scaled to the range [0,1].

### Data Transformation and Distribution Assessment

In addition to feature scaling, the distributions of each predictor variable were examined to identify skewness and non-normality, which are common in experimental datasets compiled from both laboratory work and published literature. Since several parameters, such as WRCA, WRFA, ZSF, SS, and SP, contain a considerable number of zero values (corresponding to mix designs where these constituents were not used), the dataset exhibits zero-inflation and right-skewed distributions for some variables. To address this, advanced transformation techniques, including the logarithmic and Box–Cox transformations, were initially considered. However, the target variable i.e. compressive strength exhibits a relatively narrow and stable range, with limited variation compared to the predictor variables. Therefore, transformation of the output variable was not required. Moreover, for models such as Random Forests and deep learning-based predictors, transforming input features is not strictly necessary, as these models can learn complex nonlinear patterns without requiring normality assumptions. Hence, in the present work, Min–Max normalization was adopted as the primary preprocessing strategy to ensure uniform scaling across all input variables and to improve training stability and convergence, especially for ANN/DNN-based models.

### Multicollinearity analysis

**Fig. 3:**
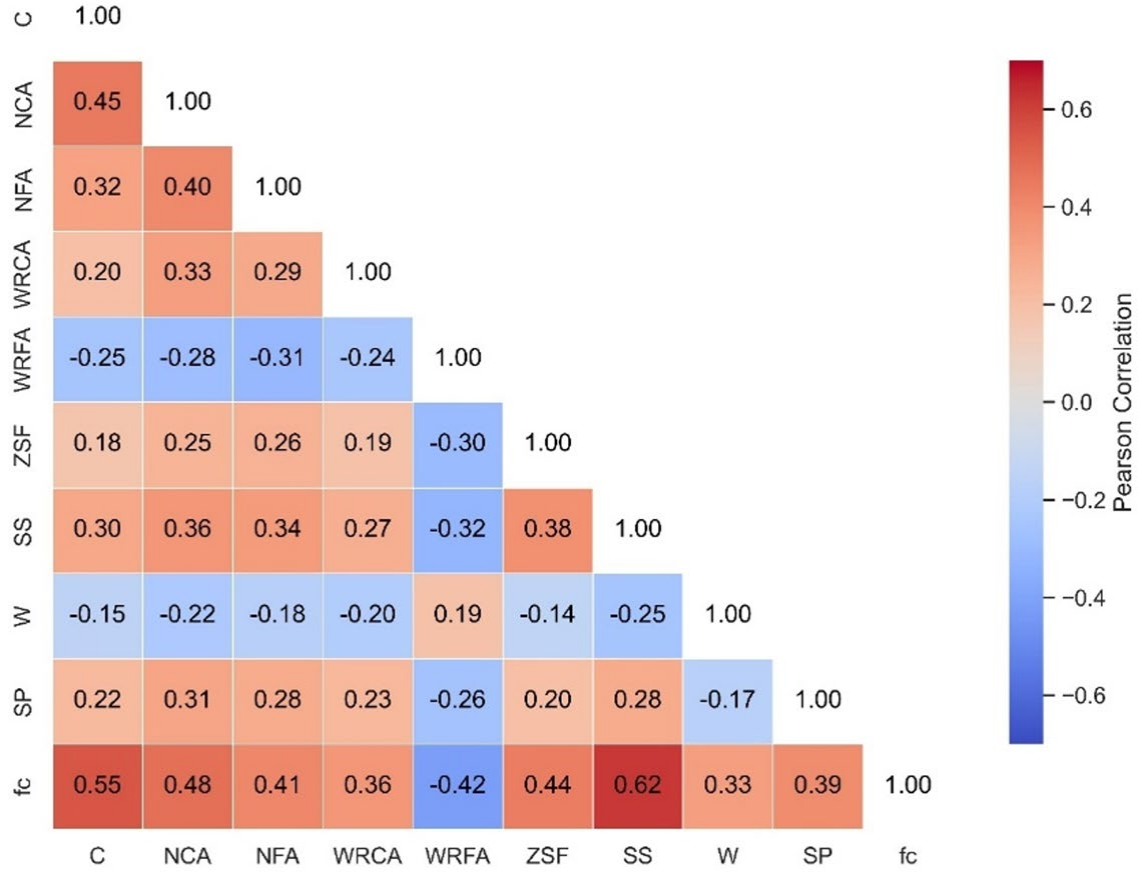
Pearson correlation matrix among input and output parameters.

Multicollinearity analysis among the input and output variables was evaluated using the Pearson correlation coefficient (r) to quantify the strength of linear relationships^57^. Figure 3 illustrates the Pearson correlation matrix provides a comprehensive understanding of the linear relationships between variables. It was observed that C, SS and SP exhibit strong positive correlations with compressive strength, with SS showing the highest coefficient (*r* = 0.62), indicating its significant contribution to strength enhancement. Moderate positive correlations were also marked for NCA, NFA, and WRCA, suggesting their supportive role in the concrete matrix. In contrast, WRFA demonstrates a moderate negative correlation (*r* = − 0.42), implying that higher WRFA content may reduce compressive strength. The other variable-W also shows a slight negative relationship with strength, consistent with typical concrete behavior where excess water dilutes the matrix.

**Fig. 4:**
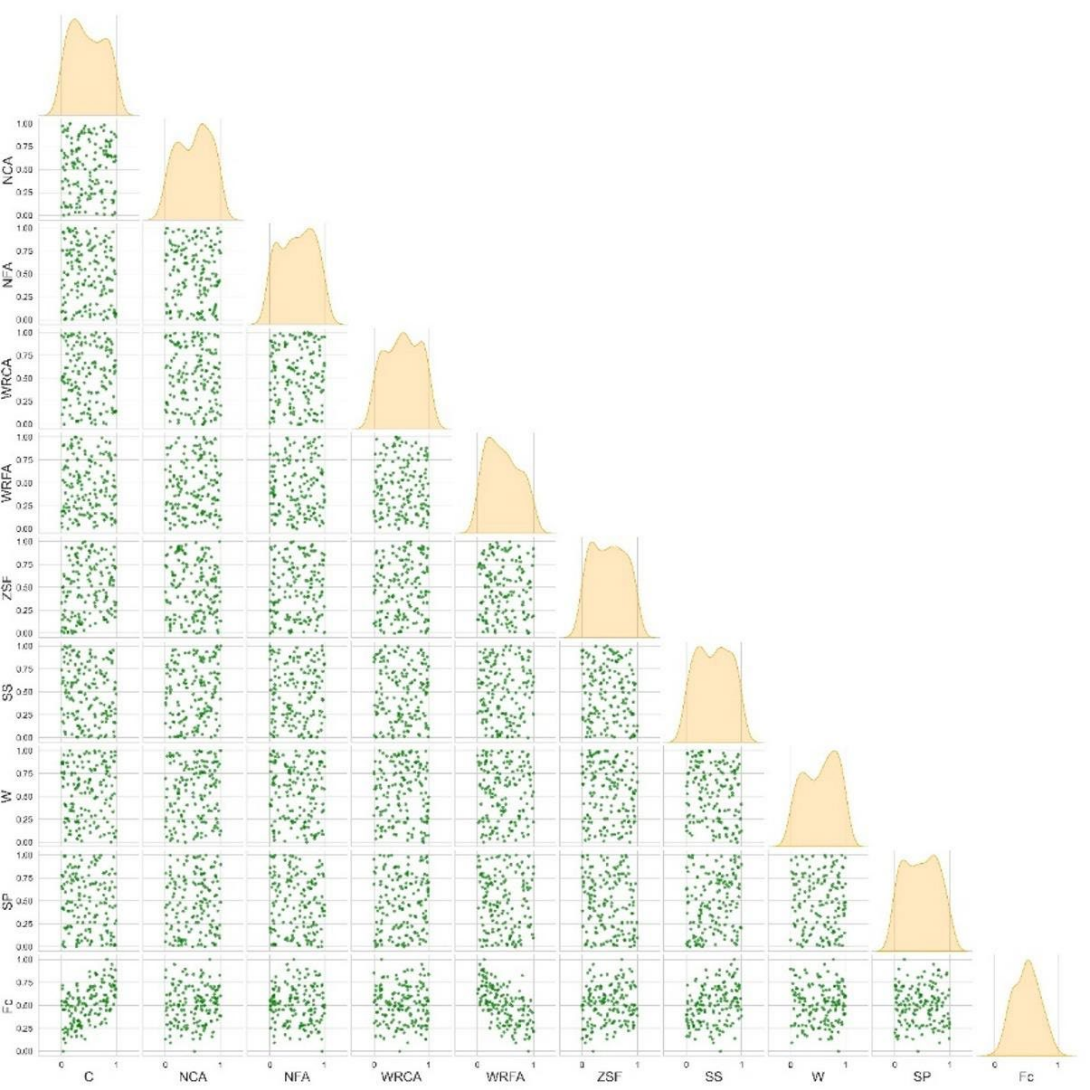
Pairwise relationships among input and output variables through Scatter plot matrix.

For more support, Fig. 4 illustrates the scatter plot of the distributi60on of each parameter. The off-diagonal scatterplots reveal the nature of pairwise interactions among variables. A noticeable upward trend was observed in C, S, SP, and fc, confirming their positive influence on strength development. In contrast, WRFA exhibits a distinct interaction pattern relative to fc, indicating that when used in optimal proportions, it can contribute positively to strength development while also enhancing the sustainability of the concrete mix. The aggregates- NCA, NFA, and WRCA display moderately uniform scatter distributions, indicating consistent and stable interactions with fc. Water content (W) presents a broader distribution with a slight downward tendency, consistent with conventional concrete behaviour and confirming the reliability of the experimental data.

## Development and selection of predictive models using machine learning techniques

In this study, the predictive models were selected to represent diverse algorithmic families commonly used for regression problems in civil engineering applications. A set of ML algorithms-kNN, RF, ANN, DNN were chosen to represent diverse learning mechanisms and model complexities, enabling a comprehensive evaluation of predictive performance. The kNN algorithm was chosen as a distance-based, non-parametric method that provides a simple benchmark and performs effectively on moderate-sized datasets. RF was selected due to its ensemble learning structure, robustness to noise, and ability to capture nonlinear relationships while reducing overfitting through bootstrap aggregation. ANNs were included as a widely adopted nonlinear modeling approach capable of learning complex input–output relationships. The DNNs were employed to evaluate the benefits of deeper architectures in capturing higher-order interactions among multiple concrete mix parameters. Finally, the hybrid FPA-DNN model was introduced to address known limitations of standalone DNNs, such as sensitivity to weight initialization and overfitting, by optimizing network parameters through a metaheuristic approach. This combination of models enables a balanced comparison across different learning mechanisms and justifies their selection for reliable compressive strength prediction.

### k-nearest neighbours

The kNN algorithm is a supervised ML algorithm used for classification and regression tasks^58^. It is a non-parametric method that makes predictions based on the k closest training samples in the feature space^27^. In kNN, the “k” refers to the number of nearest neighbours to consider. When making predictions, the algorithm identifies the kNN to a given query point and determines the class or value based on the majority or average of their labels, respectively^59^. The kNN algorithm can be used for regression problems by predicting the value of a new data point based on the values of its kNN^60^. The choice of distance function is a crucial factor in KNN regression as it directly affects the prediction accuracy. The Minkowski distance function is a widely used distance metric for continuous variables in KNN regression, as shown in Eq. (2). It is a generalized distance metric that includes both the Manhattan distance (*p* = 1) and the Euclidean distance (*p* = 2) as special cases. The target value of the new data point is then predicted by taking the average of the target values of its k nearest neighbours. The pseudocode for kNN has been provided in Table A1.

**Table A1:**
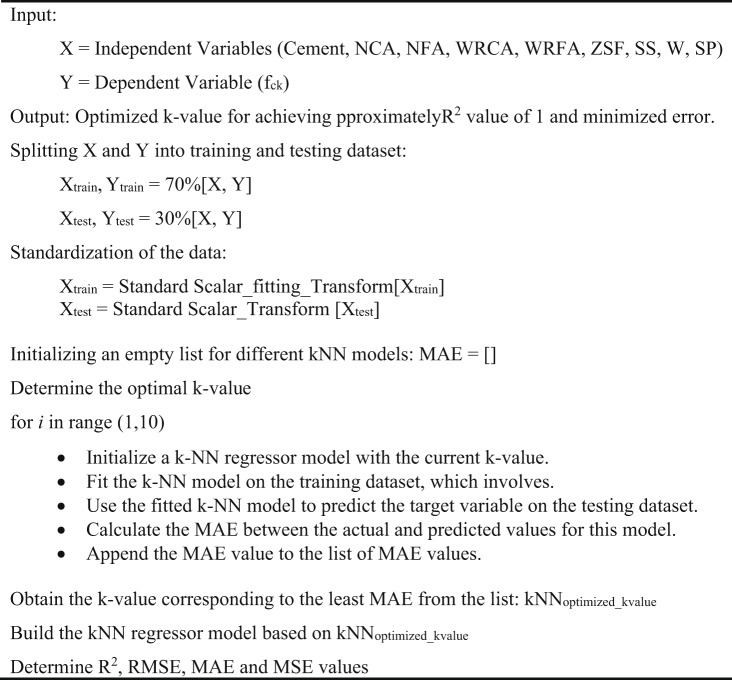
Algorithm of k-NN model used in the present study.

2 $$\:{\left(\sum\:_{i=1}^{N}{\left|\left({X}_{i}-{Y}_{i}\right)\right|}^{p}\right)}^{\frac{1}{p}}\:\:\:$$

Where, $\:{X}_{i}$ and $\:{Y}_{i}$ are the two data points in N dimension space.

### Random Forest

The RF algorithm is a supervised ML technique used for classification and regression tasks^61^. It belongs to the ensemble learning family of algorithms, where multiple ML models are trained to solve the same problem, and their results are combined to make a more accurate prediction^62^. Random Forest algorithm uses the combined output of the interlinked decision trees to yield the outcome. It can handle a large amount of data, mitigate the overfitting issues of the model, and provide high accuracy in regression modelling. The training data (denoted as T_n_) with ‘n’ numbers of training data points can be written as shown in the Eq. (3)

3 $$\:{T}_{n}=\left\{\left({x}_{1},{y}_{1}\right),\:\left({x}_{2},{y}_{2}\right),\:\left({x}_{3},{y}_{3}\right),\dots\:\dots\:\dots\:,\:\left({x}_{n},{y}_{n}\right)\right\}\:\:\:\:\:\:\:\:\:\:\:\:\:\:\:\:\left({x}_{i}\in\:{R}_{i},{y}_{i}\in\:R\right)$$

The training function contains a global observed function $\:y=a\left(x,\widehat{{R}_{n}}\right)\:,$ at the last R_n_. So, the average output obtained from decision trees is $\:\widehat{{y}_{1}}=\widehat{a}\left(x,\widehat{{R}_{1}}\right),\:\widehat{{y}_{2}}=\widehat{a}\left(x,\widehat{{R}_{2}}\right),\:\widehat{{y}_{3}}=\widehat{a}\left(x,\widehat{{R}_{3}}\right),\dots\:.\widehat{{y}_{t}}=\widehat{a}\left(x,\widehat{{R}_{n}}\right)$. Thus, the final output derived based on the input vector x is computed using Eq. (4). The pseudocode for the random forest algorithm used in this study has been provided in Table A2.

**Table A2:**
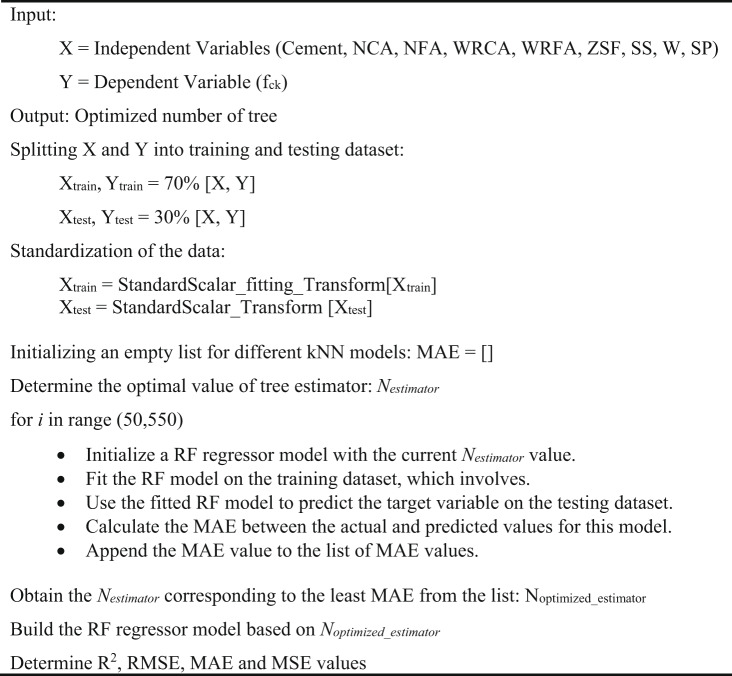
Algorithm of RF model used in the present study.

4 $$\:y=\frac{1}{k}\sum\:_{n=1}^{k}\widehat{{y}_{n}}=\:\frac{1}{k}\sum\:_{n=1}^{k}\widehat{a}\left(x,\widehat{{R}_{n}}\right)$$

### Artificial neural network

ANNs are computational models inspired by the biological structure of the human brain and are widely used for solving complex nonlinear regression and classification problems^63^. An ANN consists of interconnected processing units, known as neurons, which are arranged in an input layer, one or more hidden layers, and an output layer. Each neuron receives weighted inputs, applies an activation function, and transmits the output to neurons in the subsequent layer. Through this layered architecture, ANNs can learn intricate relationships between input variables and target outputs^64^. The output neuron can be obtained by multiplying each neuron by its corresponding weight and summing the result with the bias, as shown in Eq. (5). The choice of activation function can have a significant impact on the performance of the neural network, as different functions possess distinct properties and are better suited to specific types of problems. Equations (6), (7) and (8) represent the common activation functions for sigmoid, tanh, and ReLU (rectified linear units), respectively.

5 $$\:\:O=\sum\:_{i=1}^{i=k}{w}_{ij}\:I+{b}_{j}$$

6 $$\:f\left(x\right)=\frac{{e}^{x}-{e}^{-x}}{{e}^{x}+{e}^{-x}}$$

7 $$\:f\left(x\right)=\frac{1}{1+{e}^{-x}}$$

8 $$\:f\left(x\right)=\mathrm{max}(0,\:x)$$

Where, O is the output, w_ij_ is the assigned weights, I is the input, and bj is the bias.

### Deep Neural Network

Geoffrey Hinton proposed a deep learning theory over ANN with the help of the brain recognition process. In DNN, it mainly consists of three layers: an input layer, one or more hidden layers, and an output layer^65^. To utilize deep learning networks for concrete strength prediction, large amounts of data are required to train the network. This data typically includes information about the concrete mix design, the environmental conditions during curing, and the resulting concrete strength^66^. Once the network has been trained, it can be used to make predictions about the strength of new concrete mixes based on the input data. The pseudocode for ANN and DNN algorithms used in this present study has been combinedly provided in Table A3.

**Table A3:**
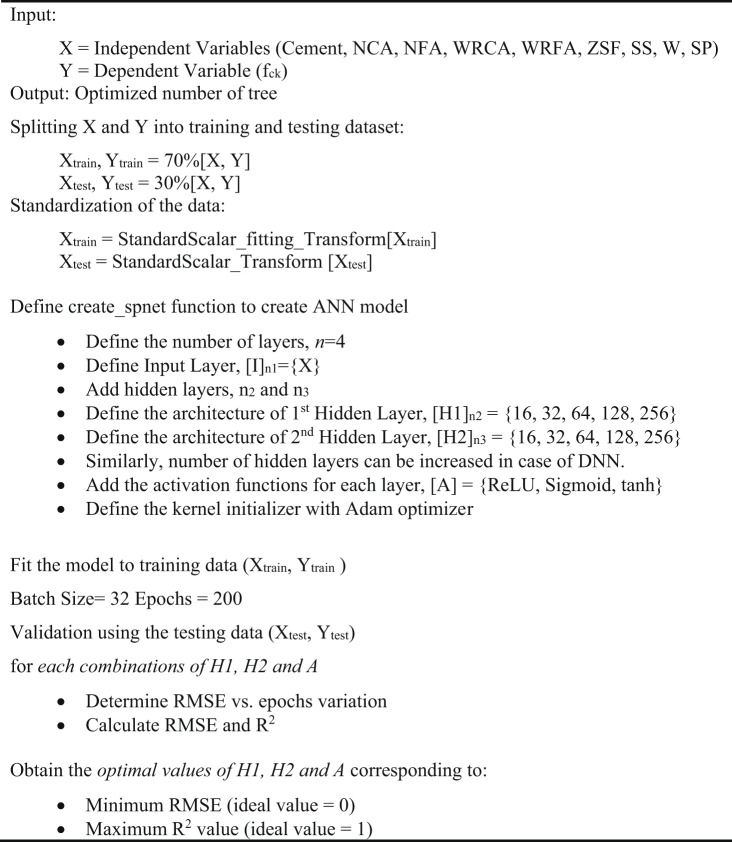
Algorithm of ANN and DNN model used in the present study.

### Deep Neural network with Flower Pollination algorithm (FPA-DNN)

A novel optimization approach is derived from the pollination phenomenon, involving the mating of flowers, leading to the generation of either new or identical flower species^67^. This process is categorized into two types:

1. Biotic (or cross-pollination), where pollen is transferred between distinct flowers, facilitated by pollinators such as bats, birds, and honeybees.
2. Abiotic or self-pollination, involving the transfer of pollen within the same species of plant flowers, with wind acting as the pollinating agent.

Observations indicate that approximately 90% of pollination occurs through biotic means, with only 10% attributed to abiotic pollination. The consistency of flower characteristics is contingent on pollen pollination. In this context, the regulation of either self or cross-pollination is influenced by a switch probability denoted as P ∈ [0.1].

FPA is a nature-inspired optimization method, making several key assumptions. These include considering the solution *Z*_*p*_ as either a flower or a pollen gamete without the need for differentiation. Bees and birds, acting as global pollinators, can fly longer distances, with their flight or jump distances following the Levy distribution. Flower constancy, representing an incremental step size denoted as “*L*” is based on the similarity or difference between two flowers. FPA involves two distinct procedures: biotic and abiotic processes, each governed by specific rules. Additionally, FPA assumes that each plant has only one flower, and each flower produces a single pollen gamete. The pseudocode for FPA-DNN algorithm used in this study has been provided in Table A4.

**Table A4:**
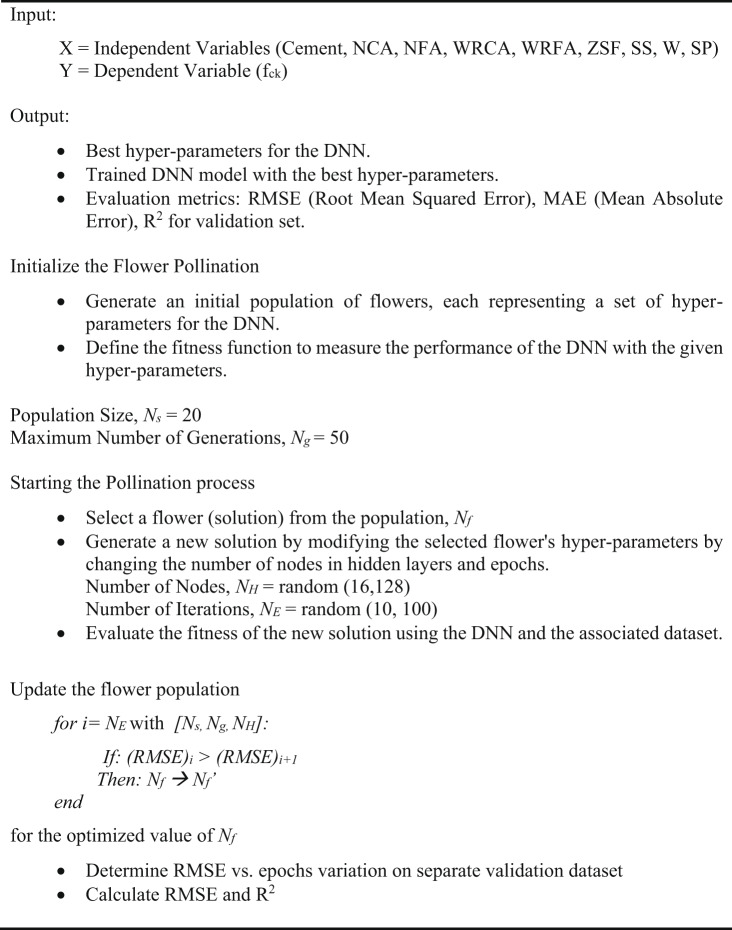
Algorithm of FPA-DNN model used in the present study.

####  1 st Rule

Global pollination in biotic pollination is characterized by pollinators, such as bees and birds, following the Levy flight for covering extensive distances, ensuring the reproduction of the most fit individuals. This global best is denoted as b_*_. The mathematical representation of global pollination is expressed through Eq. 9.

9 $$\:{Z}_{f}^{p+1}={Z}_{f}^{p}+\omega\:H\left(\lambda\:\right)({b}_{*}-{Z}_{f}^{p})$$

The variable b_*_ represents the current best solution identified among the solutions at the current iteration. Character *H* denotes the Levy flight, a 22-character sequence that simulates the varying flight distance of the insect. Equation 10 describes the flight distance, adhering to Levy’s distribution, where T > 0.

.

10 $$\:H=\frac{\lambda\:{\Gamma\:}\left(\lambda\:\right)\mathrm{sin}\left(\frac{\pi\:\lambda\:}{2}\right)}{\pi\:}\frac{1}{{T}^{1+{\lambda\:}^{{\prime\:}}}}(T>{T}_{o}>0)$$

#### 2nd Rule

Self or abiotic pollination, referred to as local pollination, is characterized by the mathematical expression of flower constancy, as represented by Eq. 11.

11 $$Z_{f}^{{p + 1}} = Z_{f}^{p} + \varepsilon (Z_{v}^{p} - Z_{s}^{p} )$$

In the context of Eq. 3, $\:{Z}_{v}^{p}$ and $\:{Z}_{s}^{p}$ represent pollens from the same plant species but originating from different flowers. Local pollination imitates a local random walk, where the parameter " $\epsilon$ " is drawn from a uniform distribution within the range of [0, 1].

#### 3rd Rule

**Fig. 5:**
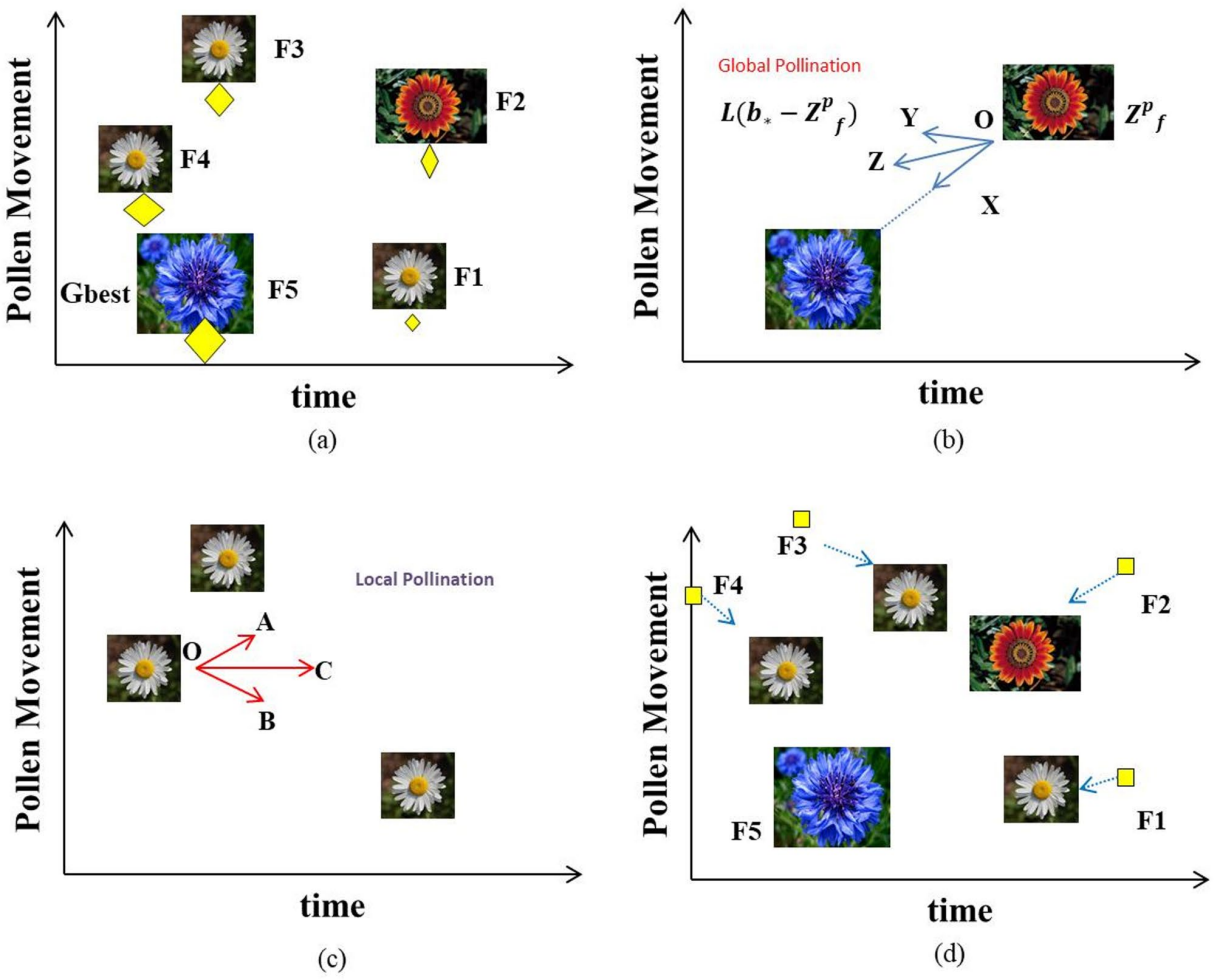
Pollen Position Update Using FPA Method which includes (a) the initialization and evaluation of fitness functions for pollens, (b) the cross-pollination process, (c) the self-pollination process, and (d) chaff updating for the subsequent iteration.

Flower pollination activity can occur either locally or globally. Local pollination takes place in nearby neighborhood flower patches at shorter distances, whereas global pollination occurs over longer distances. The determination of whether to opt for global or intensive local pollination is guided by the switch probability, denoted as P, which falls within the range [0, 1]. In many instances, the optimal value for the switch probability is observed to be maintained at 0.8 based on previous cases. The illustrative diagram of the working of PFA is shown in Fig. 5. By integrating FPA with the DNN model, the prediction accuracy has increased tremendously.

### Model Performance statistical indicators

The various statistical parameters such as mean absolute percentage error (MAPE), root mean square (RMSE), coefficient of determination (R^2^), Mean Bias Error (MBE) and Mean Absolute Deviation (MAD) to evaluate the predictive performance of model. These indicators were calculated for both the training and testing phases through the Eqs. (12)–(16) to assess the accuracy, bias, and robustness of the models in predicting the compressive strength of concrete pavements^68,72,73,71^.

12 $$\:MAPE=\:\frac{100}{n}\sum\:_{I=1}^{n}\frac{ei-pi}{ei}$$

13 $$\:RMSE=\sqrt{\frac{\sum\:_{i=1}^{n}{(ei-pi)}^{2}}{n}}$$

14 $$\:\:{\:R}^{2}=1-\left(\frac{\sum\:_{i}{\left({p}_{i}-{e}_{i}\right)}^{2}}{\sum\:_{i}{\left({e}_{i}\right)}^{2}}\right)$$

15 $$\:MBE=\frac{1}{n}\sum\:_{i=1}^{n}({e}_{i}-{p}_{i})$$

16 $$\:MAD=\frac{1}{n}\sum\:_{i=1}^{n}\left|{e}_{i}-{p}_{i}\right|$$

where, $\:{e}_{i}$ is the experimental value, $\:{p}_{i}$ is the predicted value & n is the total no. of concrete samples.

### SHAP interpretability analysis

To evaluate the contribution of individual input variables toward the predicted compressive strength, SHAP analysis was performed on the optimized FPA-DNN model. SHAP provides a unified framework for interpreting complex ML models by quantifying the marginal contribution of each feature to the model output^72^. This analysis provides a clear understanding of how variations in input parameters affect the predicted strength values, thereby enhancing the explainability of the developed model^73^. Mathematically, SHAP values for feature j were calculated using Eq. (17)

17 $$\:{\phi\:}_{j}={\sum\:}_{S\subseteq\:N\left\{j\right\}}\frac{\left|S\right|!\left(\left|N\right|-\left|S\right|-1\right)!}{\left|N\right|!}[f\left(S\bigcup\:\left\{j\right\}\right)-f\left(S\right)]$$

In this formulation, $\:{\phi\:}_{j}$ is the SHAP value of feature *j*, $\:N$ is the set of all input features, $\:S\:$is the any subset of features not containing $\:j$, and $\:f\left(S\right)$ is the model output.

## Results and discussion

### Compressive strength

**Fig. 6:**
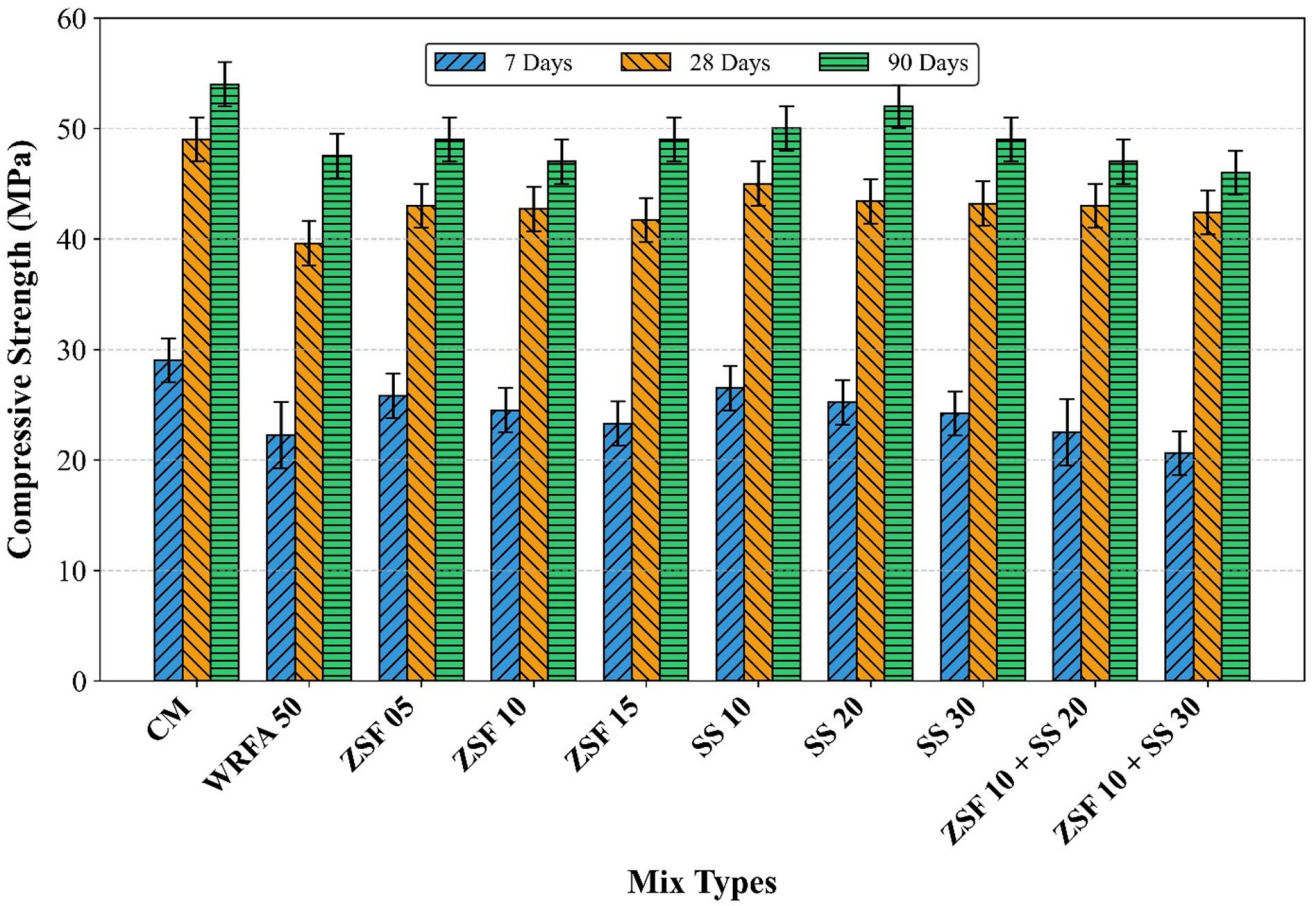
Influence of Compressive strength containing WRFA.

The results of compressive strength in different trial mixes at 7, 28 and 90 days of curing age are presented in Fig. 6. From the available literature, incorporation of 50% of WRFA was noted as a reference compared to other trial mixes. After 7 days of curing, the compressive strength of various mixes was observed to be reduced by 29.6% after incorporating the WRFA50. The addition of SCMs has marginally decreased strength. However, the addition of SS 20 as a partial substitute has the least impact among other SCMs, lowering the compressive strength of concrete by 14%. The test conducted at 28 days revealed a similar trend of a decrease in strength with the addition of WRFA, along with the considered SCMs. The analysis of long-term strength, i.e., 90 days, indicates that ZSF10, SS10, SS20, and SS30 are the best substitutes, with minimal effects on compressive strength (8.9%, 6.6%, 6.3%, and 8.4%, respectively). Interestingly, upon replacement by SS20, a slight reduction in compressive strength was noted, yet the desired strength criteria were still achieved. This reduction in compressive strength values with different proportions indicates the presence of asphalt coating on the surrounding RAP aggregates and cement mortar^74^. Moreover, the combination of ZSF and SS does not have a great influence on compressive strength. The result from the present study indicates that 20% of SS could be utilized in the construction of rigid pavement incorporating WRFA.

### Flexural strength

**Fig. 7:**
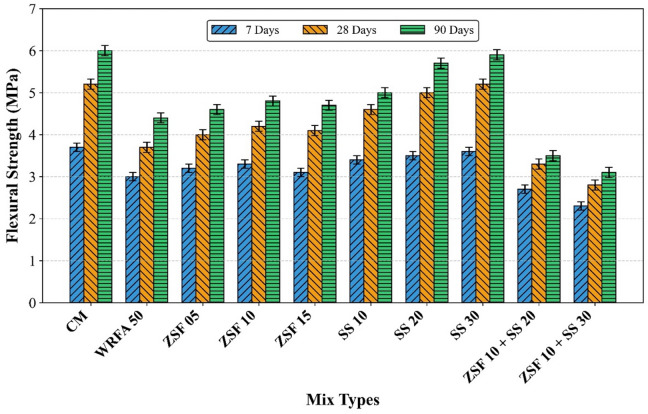
Influence of Flexural Strength Containing WRFA.

The effect of WRFA on the flexural strength of concrete is shown in Fig. 7. During the initial days of the curing period, the inclusion of WRFA50 tends to decrease considerably. For instance, the addition of ZSF (5, 10 & 15%) and SS (10, 20 & 30%) shows a decrease in strength of 47.9%, 34.2%, 5.2%, 19.2%, 14.9%, and 7.4%, respectively. Moreover, the combination with ZSF 10 at SS 20 and 30% shows a high reduction rate of 57.4% and 80.6%, respectively. Meanwhile, the study at 28 days of curing reveals that the addition of SCMs decreases the strength when used in conjunction with WRFA50. The asphalt layer, which offers inadequate adhesion between the aggregate surface and the cementitious mortar matrix, is responsible for the decrease in concrete strength^15^. Interestingly, the combination of ZSF 10 + SS20 mix as a partial replacement of OPC yields an increase in strength of 5.8%, while in the case of ZSF 10 + SS30, it remarkably decreases strength by 81.5% compared to the control mix. It was observed that the incorporation of SCMs significantly decreases the strength; however, SS 20 and SS 30 mixes have the least effect on strength reduction, at 3% and 6%, respectively. So, SS20 mix can be recommended as an optimal partial substitute for cement with WRFA in achieving the desired flexural strength.

### Split tensile strength

**Fig. 8:**
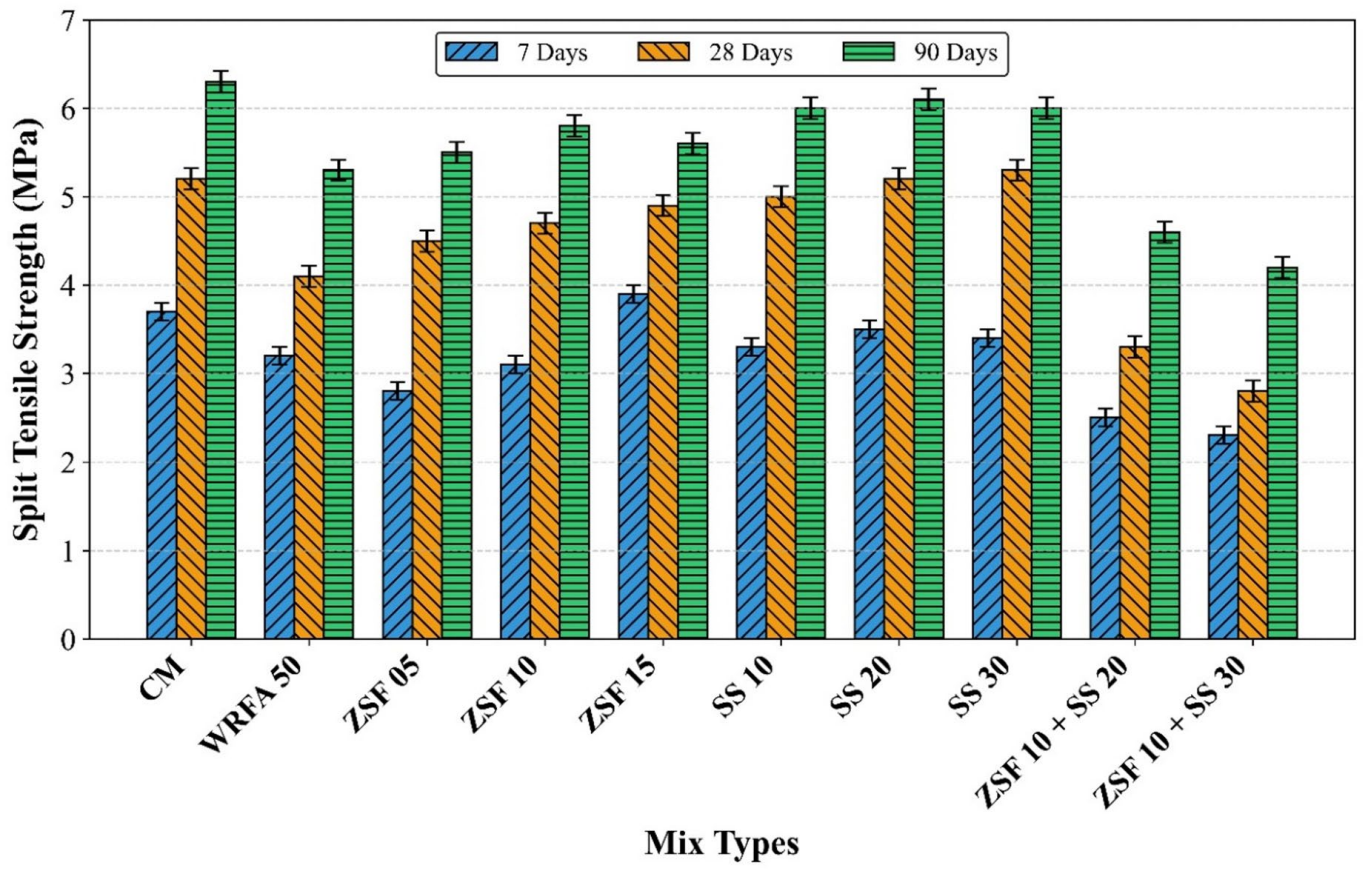
Influence of the Tensile strength of fine RAP containing WRFA.

The splitting tensile strength of RAP concrete mixes incorporating WRFA at 7, 28, and 90 days of the curing period is illustrated in Fig. 8. Like the compressive and flexural strength, the split tensile strength decreased with the incorporation of WRFA as compared to the control mix. With the addition of 50%WRFA, a 16.13% decrease was observed in tensile strength at 7 days of the curing period compared to the control mix. The optimal proportion observed corresponds to ZSF15, with a 7.7% increase in tensile strength compared to the control mix. Therefore, for the early strength replacement of Portland cement with ZSF15, it is recommended to be used in conjunction with WRFA. Further studies conducted in 28 days reveal a decreasing trend in tensile strength with the incorporation of WRFA50, resulting in a 31.7% decrease in strength. This reduction in tensile strength was attributed to the film barrier surrounding the RAP, which led to the formation of an interfacial transition zone (ITZ) in the concrete^15^.

It was also found that the mix with SS20 and SS30 has an insignificant loss of 3.8% in strength. After evaluating the long-term strength, it can be concluded that the reduction in tensile strength due to the replacement of WRFA50 was 21.2%. However, it is interesting to note that SS 20 outperforms all the combinations of admixture, with the least reduction rate of 3.3%. So, the findings recommended the use of SS 20 as a partial replacement for OPC with the reference mix. On the other hand, the combining effect i.e. 10% ZSF with 20% SS seems to be reduced at 50%, 58.8% and 36.9% as compared to the reference mix (WRFA50) at respective curing ages, whereas further addition of 30% SS the tensile strength lowered by 40.9%, 74.8%, and 59.9% at 7, 28, and 90 days respectively. This occurred due to the presence of excessive tiny voids in the concrete mixes, which created a poor bond between the asphalt and the cement mortar matrix containing fine aggregates.

### Micro-structure observations

**Fig. 9:**
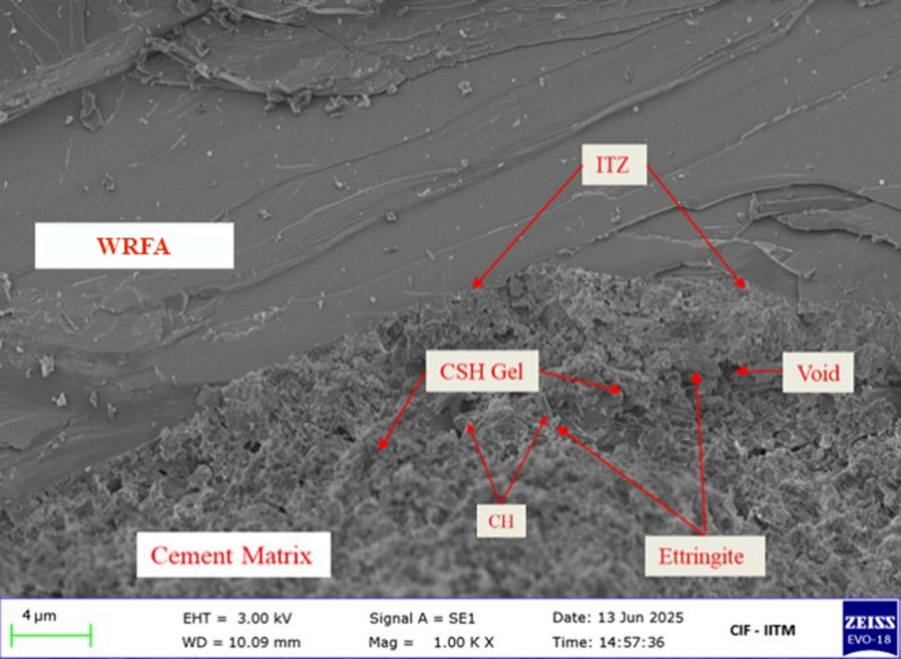
SEM microstructure image of the optimal concrete mix (WRFA50 + SS20).

Figure 9 presents the scanning electron microscope (SEM) microstructure image of the optimal concrete mix WRFA50 + SS20. The micrographs reveal a relatively dense and compact cementitious matrix with reduced pore connectivity and improved interfacial bonding between the aggregates and the paste. The presence of SS contributes to secondary hydration reactions, leading to the formation of additional calcium silicate hydrate (C–S–H) gel, which enhances matrix densification. The ITZ between the WRFA particle and the cement paste appears compact and well bonded, indicating improved paste–aggregate interaction due to effective washing of WRFA and the contribution of SS content^74^. Negligible voids were detected, suggesting reduced porosity compared to conventional recycled aggregate concrete. The presence of calcium hydroxide (CH) and ettringite confirms the hydration and secondary reactions, while their controlled distribution indicates matrix densification. These microstructural characteristics provide clear evidence supporting the improved compressive strength performance observed for the optimal mix and validate the experimental findings of the study.

### Hyperparameter selection of ML model

**Fig. 10:**
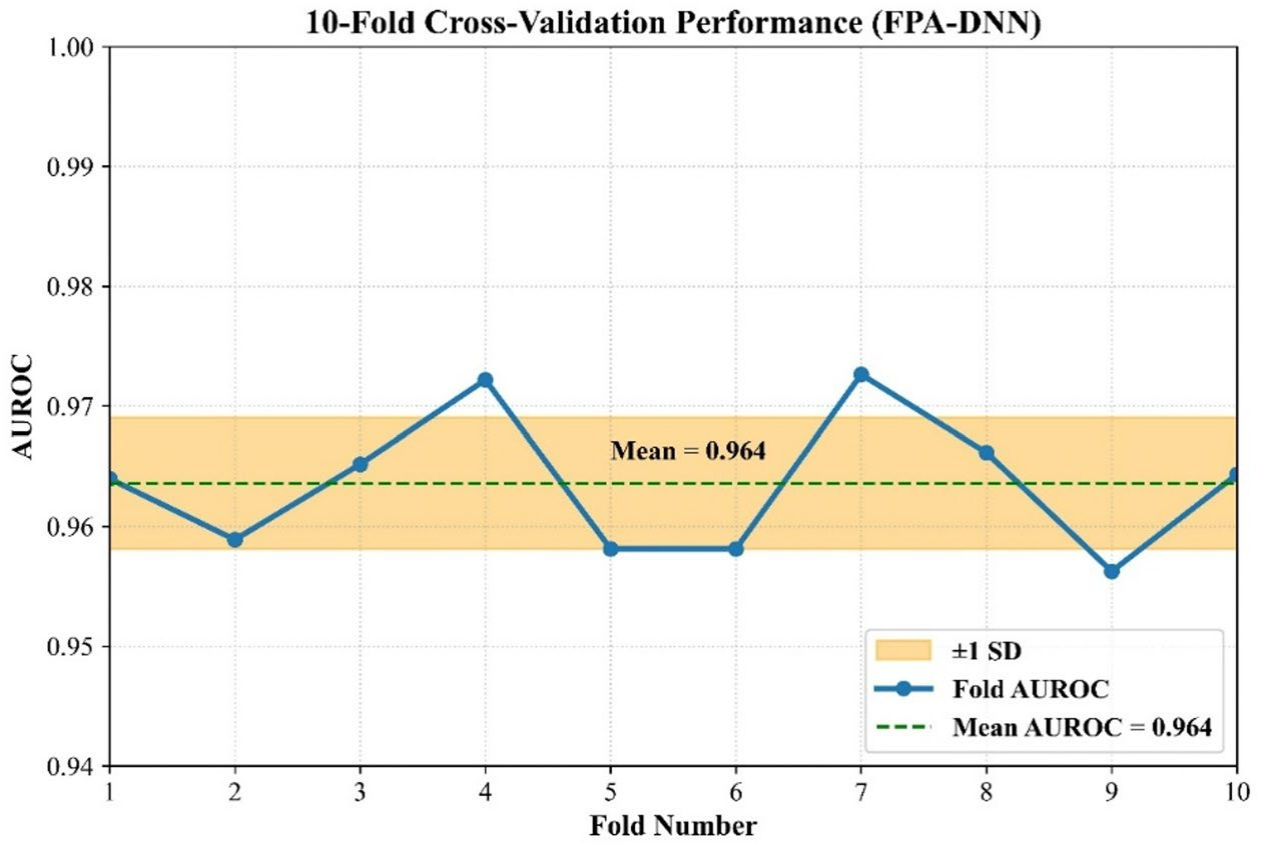
Result of 10-fold cross-validation of the proposed model.

Hyperparameter selection is a crucial step in ensuring stable model training and reliable predictive performance. The performance of ML models is strongly influenced by the choice of hyperparameters, and improper tuning may lead to suboptimal predictions or overfitting^75,76^. In this study, hyperparameters for all the ML models were optimized using an empirical grid search strategy combined with 10-fold cross-validation (k = 10). This systematic approach enabled the evaluation of multiple hyperparameter combinations and the selection of configurations that provided the best predictive performance^77,78^. By rigorously tuning key hyperparameters within predefined ranges, the procedure enhanced model robustness and ensured accurate and reliable prediction outcomes^79^. Multiple combinations of hyperparameters were evaluated for each model and the optimal configuration was selected based on cross-validated performance metrics. This approach ensured that model tuning was not reliant on default parameter settings and minimized the risk of overfitting. The use of cross-validation further enhanced the robustness of the selected hyperparameters by assessing model stability across different data splits. Table 4 summarizes an optimal hyperparameter configuration determined for each model through iterative evaluation. The result of model validation using the 10-fold cross-validation technique is shown in Fig. 10.

**Table 4:** Optimized hyperparameter configuration.

Model	Hyperparameter	Search Range	Optimal Value
kNN	Number of neighbors (*k*)	3–20	7
	Distance metric	Euclidean, Manhattan, Minkowski	Euclidean
	Weight function	Uniform, Distance	Distance
RF	*n_estimators*	100–1000	500
	Maximum depth	5–50	25
	Minimum samples split	2–10	4
	Maximum features	sqrt, log2, None	Sqrt
ANN	Hidden layer sizes	[(8,8), (16,8), (32,16,8)]	(16, 8)
	Activation function	ReLU, Tanh, Sigmoid	ReLU
	Learning rate	0.0001–0.01	0.001
	Optimizer	Adam, RMSprop, SGD	Adam
DNN	Number of hidden layers	3–6	4
	Neurons per layer	32–256	[128, 64, 32, 16]
	Dropout rate	0.1–0.5	0.2
	Batch size	16–128	32
	Epochs	50–500	200
FPA-DNN	Population size	10–50	25
	Pollination probability (*p*)	0.6–0.9	0.8
	Levy flight coefficient (*λ*)	0.5–2.0	1.5
	Iterations	50–300	200

### Comparative evaluation of ML models with statistical uncertainty analysis

**Fig. 11:**
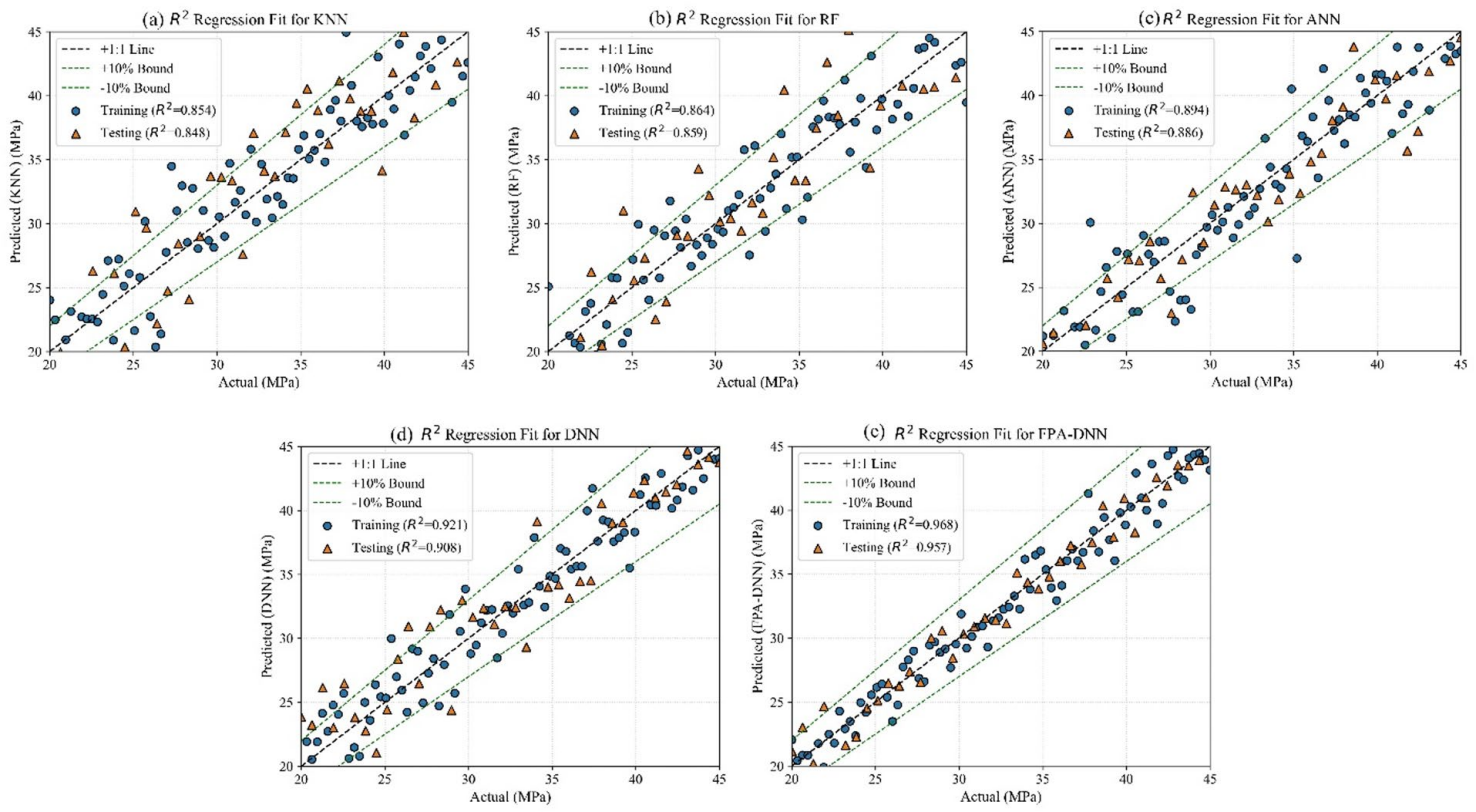
Comparison of predicted versus actual values of compressive strength using regression plot analysis.

The predictive performance of all developed ML models was evaluated using regression plot analysis, error distributions, and multiple statistical indicators obtained from 10-fold cross-validation with 95% confidence interval (CI). Figure 11 illustrates the regression fit between the predicted and actual compressive strength values. The performance of different ML models using 10-Fold Cross-Validation (Mean ± 95% CI) is provided in Table 5. The KNN and RF models exhibit moderate predictive capability, with R² values ranging between 0.85 and 0.87 across training and testing datasets. This is consistent with their relatively higher RMSE and MAPE values (RMSE ≈ 4.8–5.1 for KNN and 4.6–4.9 for RF), confirming that these models capture general patterns in the dataset with complex nonlinear interactions^80^. The ANN model demonstrates improved performance, supported by higher training and testing R² values of 0.90 and 0.89, respectively. The regression plots show tighter clustering around the ideal line, and the relative error distribution has a narrower spread than for KNN and RF. The reduction in RMSE (≈ 4.1–4.5) and MAPE (≈ 6.5–6.9%) highlights the ANN’s enhanced capability to learn nonlinear relationships. The DNN model further improves prediction quality, as reflected in its R² values (0.93 in training and 0.91 in testing) and reduced prediction error. The corresponding RMSE and MAD values (3.68–4.01 and 2.72–3.05, respectively) confirm a significant enhancement in prediction consistency.

**Fig. 12:**
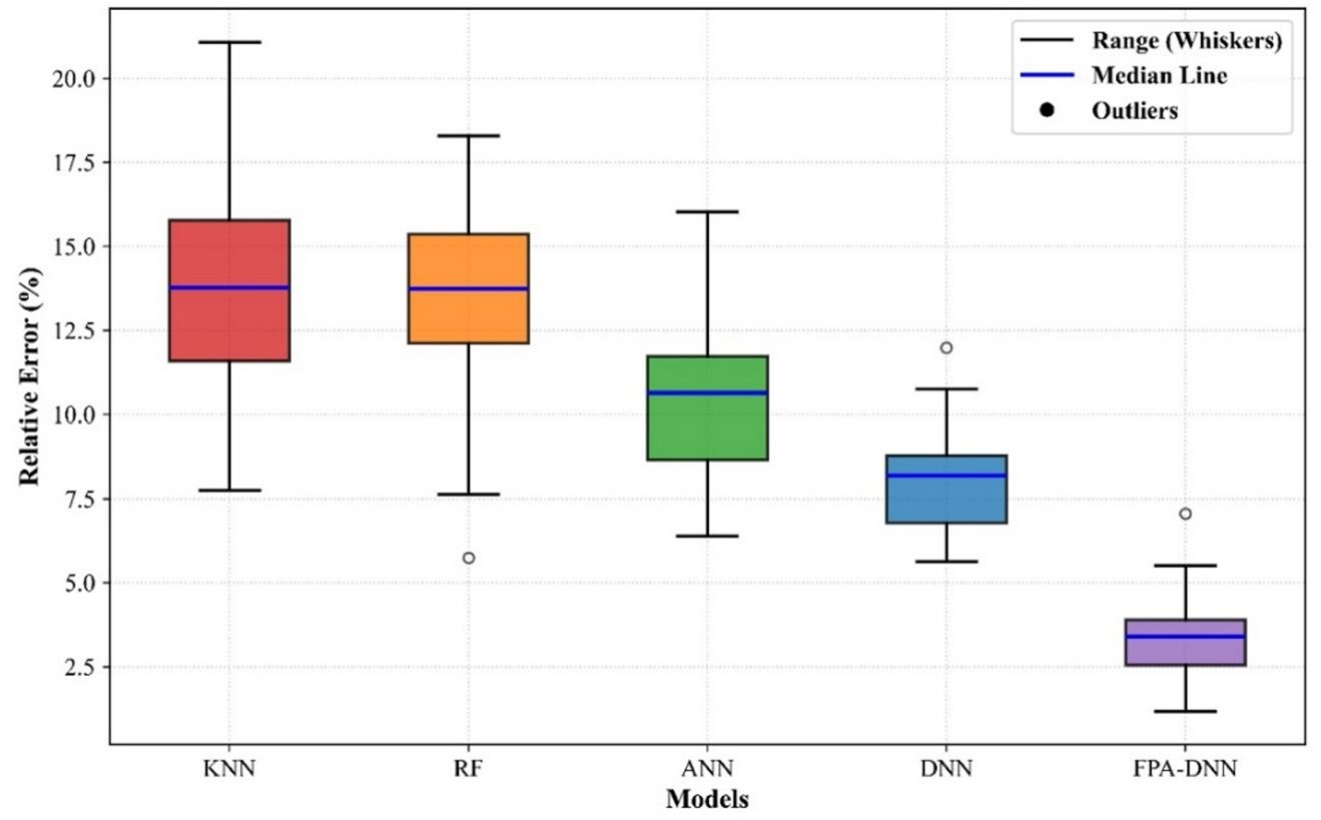
Box plot of relative error measurement.

The regression plots show highly concentrated data points around the 1:1 line for both training and testing phases in case of optimized FPA-DNN model. This showed a highest R² values (0.97 training and 0.96 testing) and the lowest error indices, including RMSE (2.42–2.87), MAPE (3.91–4.38%), and MAD (1.61–1.94). These results clearly demonstrate that the FPA–DNN optimized model exhibits superior predictive accuracy, thereby enhancing the reliability and robustness of compressive strength prediction. Figure 12 presents the boxplot distribution of relative prediction errors for the developed ML models. It was marked that the kNN and RF models exhibit wider interquartile ranges and higher median errors, indicating greater variability and reduced prediction stability. The ANN model shows moderate improvement with a narrower error spread, while the DNN model further reduces both the median error and dispersion. In contrast, the FPA–DNN model demonstrates the lowest median relative error and the smallest interquartile range, with minimal outliers, indicating superior accuracy and robustness. This confirms that optimization of the DNN using the FFA effectively enhances prediction consistency and minimizes error variability compared to conventional and standalone deep learning models.

**Table 5:** Performance of different ML models using 10-Fold CV (Mean ± 95% CI).

Model	Phase	*R*²	RMSE	MAPE	MBE	MAD
KNN	Training	0.86±0.008	4.82±0.10	7.95±0.15	0.41±0.038	3.76±0.08
	Testing	0.85±0.010	5.14±0.13	8.42±0.19	0.57±0.048	4.05±0.10
RF	Training	0.87±0.008	4.58±0.09	7.34±0.14	0.34±0.034	3.52±0.08
	Testing	0.86±0.010	4.96±0.12	7.89±0.18	0.46±0.045	3.79±0.10
ANN	Training	0.90±0.007	4.12±0.09	6.52±0.13	0.26±0.032	3.18±0.08
	Testing	0.89±0.009	4.56±0.11	6.93±0.16	0.39±0.041	3.46±0.10
DNN	Training	0.93±0.006	3.68±0.08	5.84±0.11	0.18±0.026	2.72±0.07
	Testing	0.91±0.008	4.01±0.10	6.29±0.14	0.31±0.036	3.05±0.09
FPA-DNN	Training	0.97±0.004	2.42±0.07	3.91±0.10	0.07±0.015	1.61±0.05
	Testing	0.96±0.006	2.87±0.09	4.38±0.13	0.12±0.021	1.94±0.07

**Fig. 13:**
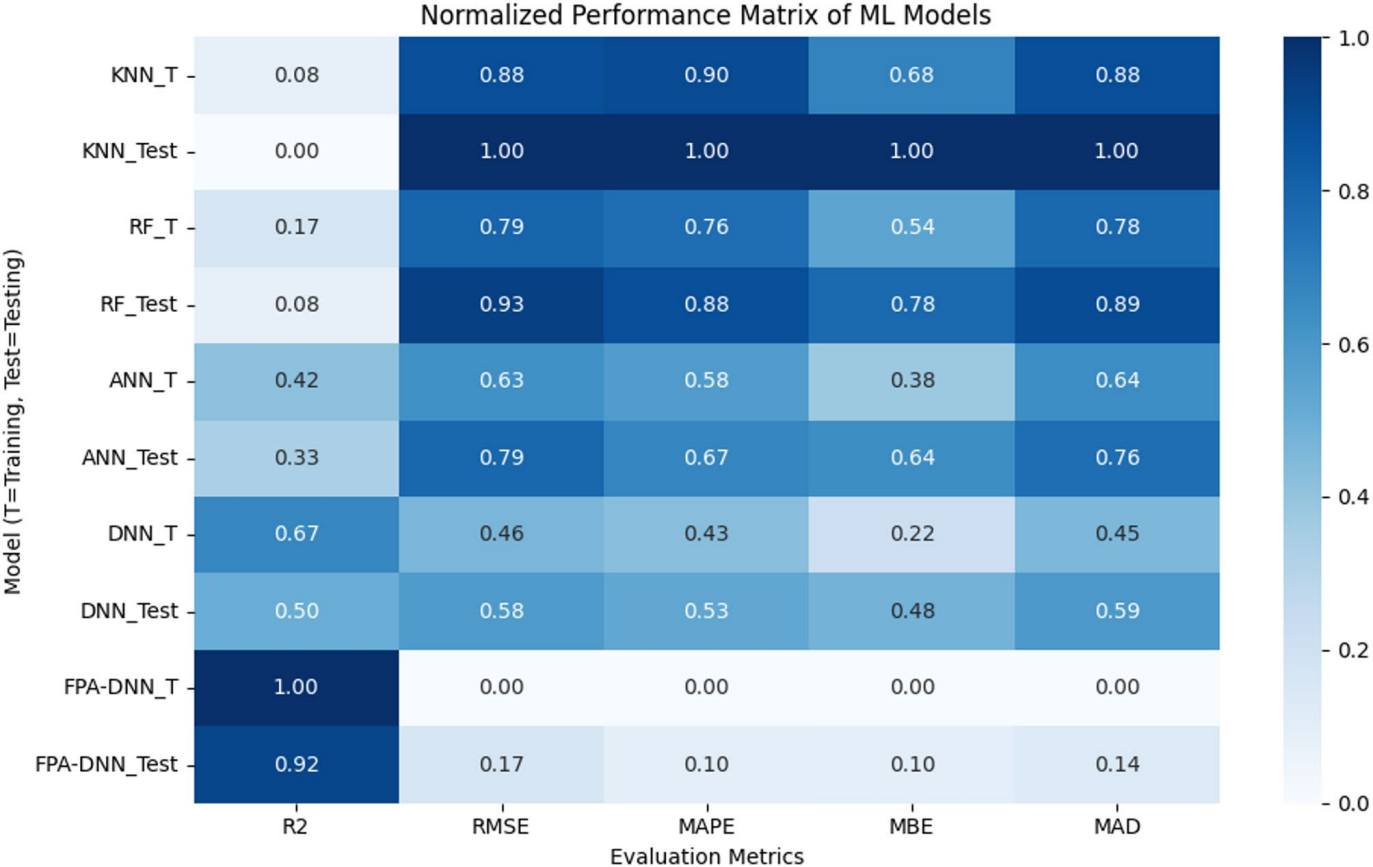
Accuracy Matrix showing the performance of prediction models.

Figure 13. depicts the accuracy matrix showing the performance of all the considered prediction models. It was marked that FPA-DNN model consistently attains the highest normalized accuracy, indicated by the strongest intensity in the heatmap, reflecting its enhanced learning efficiency and the significant improvement achieved through optimization. The DNN and ANN models exhibit moderately high accuracy levels, demonstrating reliable predictive ability but still falling short of the optimized framework. On the other hand, RF shows lower accuracy values, while kNN records the lowest among the five models, as seen from the lighter shades.

### Cross-Source validation and generalization assessment

To evaluate the robustness of the proposed ML framework under potential covariate shift, a leave-source-out validation strategy was adopted. Since the dataset consists of both laboratory-generated samples and data collected from external literature, differences in material sources, experimental procedures, and testing conditions may introduce distributional discrepancies between data sources^81^. Assessing model performance across these domains is therefore necessary to obtain a realistic estimate of generalization capability.

Two complementary cross-source experiments were conducted. In the first scenario, the models were trained exclusively using the laboratory-generated dataset and subsequently tested on samples obtained from external literature. In the second scenario, training was performed using the literature-based dataset, while the laboratory data were reserved exclusively for testing. In both cases, identical preprocessing steps, hyperparameter configurations, and evaluation metrics were maintained to ensure consistency. The results indicate a reduction in prediction accuracy compared to within-source validation, which is expected due to covariate shift between the data sources. Nevertheless, the proposed FPA-DNN model consistently demonstrated better generalization performance than the conventional ML and standalone DNN models across both validation scenarios. This suggests that the hybrid optimization strategy improves model robustness when exposed to unseen data distributions. However, the observed performance degradation highlights the influence of source-specific variability and underscores that the predictive reliability is strongest within the range of material properties and conditions represented in the training data. Accordingly, the generalization claims of the present study are limited to the observed data domain, and extrapolation to substantially different material systems or construction conditions should be approached with caution. Future studies incorporating larger, more diverse multi-source datasets are recommended to further enhance cross-domain generalization.

## Effect of White Gaussian Noise on the performance of proposed models

To assess the robustness of the developed ML regression models under realistic uncertainty, a noise-robustness test was conducted by introducing additive white Gaussian noise (AWGN) only into the output variable, i.e., compressive strength (), while keeping all input features unchanged. This procedure simulates practical disturbances such as experimental variability, sensor-induced noise, and minor fluctuations during strength testing, without modifying the original feature space. The noisy response $\:{f}_{c,\:noisy}^{i}$ was generated using Eq. 18.

18 $$\:{f}_{c,\:noisy}^{i}={f}_{c}^{i}+{\xi\:}_{i}$$

Where, $\:{f}_{i}$ represents the original response, $\:p$denotes the noise variance, and $\:{\xi\:}_{i}\sim\:N(0,{\sigma\:}^{2})$ is a gaussian random variable for the $\:{i}^{th}$sample. This ensures that the input feature space remains unchanged while controlled white noise contaminates the output, enabling a consistent evaluation of noise sensitivity. The noise standard deviation was scaled relative to the variability of the dataset as $\:\sigma\:=p\times\:std\left({f}_{c}\right)$; where std(fc) = 3.29 MPa (Table 3), and was selected as 0.01, 0.05, 0.10, and 0.20. These noise levels correspond to ≈0.033, 0.165, 0.329, and 0.658 MPa, respectively, which represent approximately 0.1%, 0.4%, 0.8%, and 1.6% of the mean compressive strength ($\:\overline{{f}_{c}}$=41.487 MPa).

To capture the stochastic effect of noise, 50 independent Monte-Carlo trials were performed for each noise level and for each model. In each trial, a new noisy target vector was generated, the models were trained using the same learning framework, and the predictive performance was evaluated. The final results are reported as mean ± standard deviation of the regression performance metrics across the 50 trials (Table 6).

**Table 6:** Noise Robustness Performance of ML Models.

Model	*p*	*R*²	RMSE	MAPE	MBE	MAD
kNN	0.00	0.850±0.006	5.14±0.10	8.42±0.18	0.57±0.05	4.05±0.09
	0.01	0.846±0.007	5.20±0.12	8.53±0.20	0.60±0.06	4.12±0.10
	0.05	0.832±0.009	5.42±0.14	8.88±0.23	0.66±0.07	4.28±0.12
	0.10	0.807±0.013	5.78±0.18	9.45±0.30	0.74±0.08	4.56±0.12
	0.20	0.752±0.020	6.49±0.25	10.58±0.42	0.89±0.11	5.10±0.22
RF	0.00	0.860±0.006	4.96±0.09	7.89±0.17	0.46±0.05	3.79±0.09
	0.01	0.857±0.007	5.02±0.11	8.01±0.19	0.49±0.05	3.86±0.10
	0.05	0.845±0.009	5.20±0.13	8.35±0.22	0.55±0.06	4.01±0.11
	0.10	0.821±0.012	5.51±0.17	8.88±0.28	0.63±0.08	4.25±0.14
	0.20	0.773±0.019	6.12±0.24	9.95±0.40	0.78±0.10	4.78±0.20
ANN	0.00	0.890±0.005	4.56±0.09	6.93±0.16	0.39±0.04	3.46±0.09
	0.01	0.887±0.006	4.62±0.10	7.02±0.18	0.41±0.05	3.52±0.10
	0.05	0.876±0.008	4.78±0.12	7.31±0.21	0.46±0.06	3.67±0.11
	0.10	0.853±0.011	5.09±0.16	778±0.28	0.54±0.07	3.92±0.14
	0.20	0.808±0.017	5.68±0.23	8.72±0.38	0.69±0.10	4.39±0.19
DNN	0.00	0.910±0.004	4.01±0.08	6.29±0.14	0.31±0.004	3.05±0.08
	0.01	0.907±0.005	4.07±0.09	6.38±0.15	0.33±0.04	3.10±0.09
	0.05	0.896±0.007	4.24±0.11	6.62±0.18	0.38±0.05	3.22±0.10
	0.10	0.875±0.01	4.56±0.15	7.02±0.25	0.46±0.07	3.45±0.13
	0.20	0.833±0.016	5.18±0.22	7.83±0.36	0.61±0.10	3.88±0.19
FPA-DNN	0.00	0.960±0.003	2.87±0.06	4.38±0.12	0.12±0.02	1.94±0.06
	0.01	0.958±0.003	2.93±0.07	4.46±0.13	0.13±0.02	1.99±0.06
	0.05	0.949±0.005	3.12±0.13	4.69±0.16	0.16±0.03	2.12±0.08
	0.10	0.936±0.008	3.43±0.13	5.08±0.22	0.22±0.04	2.36±0.11
	0.20	0.907±0.013	4.05±0.20	5.86±0.33	0.35±0.07	2.85±0.18

**Fig. 14:**
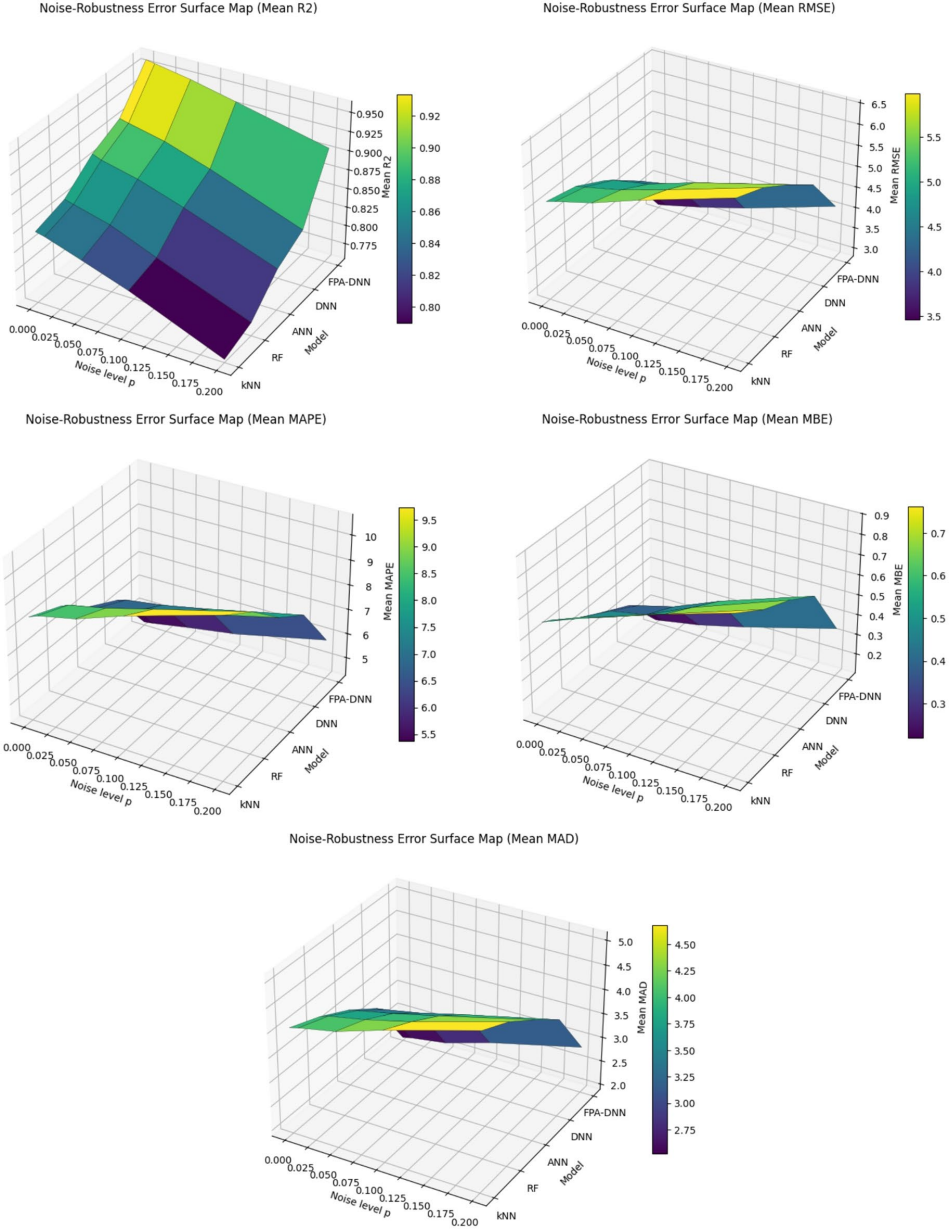
Noise Robustness Error Surface Map of different performance evaluation parameters.

**Fig. 15:**
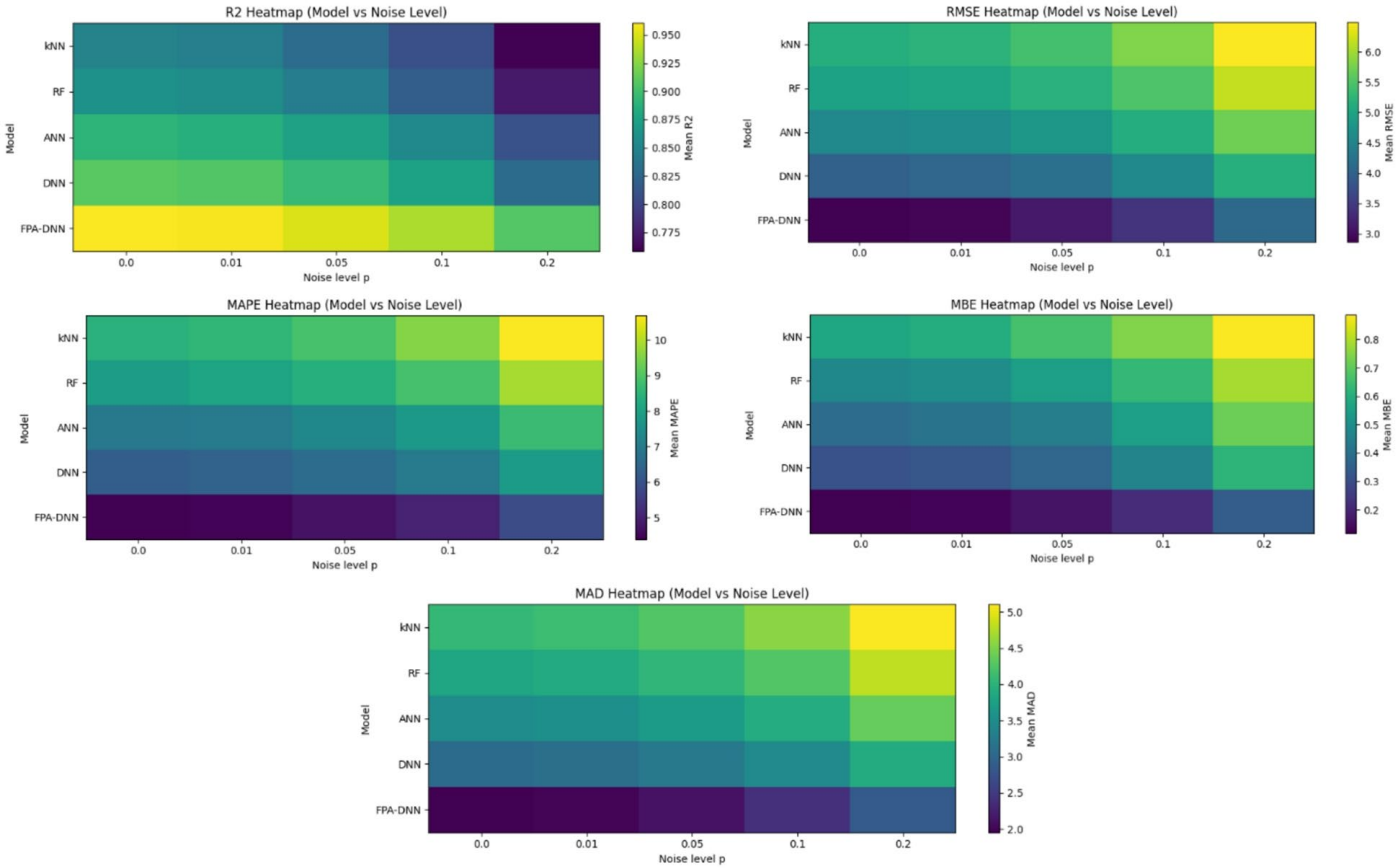
Performance Evaluation through Heatmap for Model versus Noise Level.

Figures 14 and 15 present a comprehensive noise-robustness assessment of all developed regression models under additive white Gaussian noise (AWGN). As shown in Fig. 14, the 3D error-surface maps clearly indicate a progressive degradation in model performance with increasing noise variance, reflected by a reduction in R² and simultaneous increase in error-based indicators (RMSE, MAPE, MAD, and MBE). The degradation is most pronounced for the conventional models (kNN and RF), which exhibit steeper slopes in the error surfaces, confirming their higher sensitivity to output perturbations. In contrast, deep learning models (ANN and DNN) show a smoother and more gradual decline, indicating improved resilience under noisy response conditions. Among all models, the optimized FPA–DNN consistently demonstrates the flattest surface trend across all evaluation parameters, retaining the highest R² and the lowest error magnitudes even at the maximum noise level (*p* = 0.20). This behaviour confirms that the FPA-based optimization improves the generalization stability of the DNN by reducing noise-induced bias and dispersion. The same trend is further validated in Fig. 15, where the heatmap comparison provides a compact visualization of the model ranking across noise levels. The heatmap shows clear performance separation, with kNN and RF shifting rapidly toward poorer error zones as noise increases, whereas FPA–DNN remains within the best-performing region across all noise conditions. Overall, the combined interpretation of Figs. 14 and 15 confirms that the proposed FPA–DNN model offers superior robustness and reliability for compressive strength prediction under realistic uncertain measurement environments.

### Sensitivity analysis

**Fig. 16:**
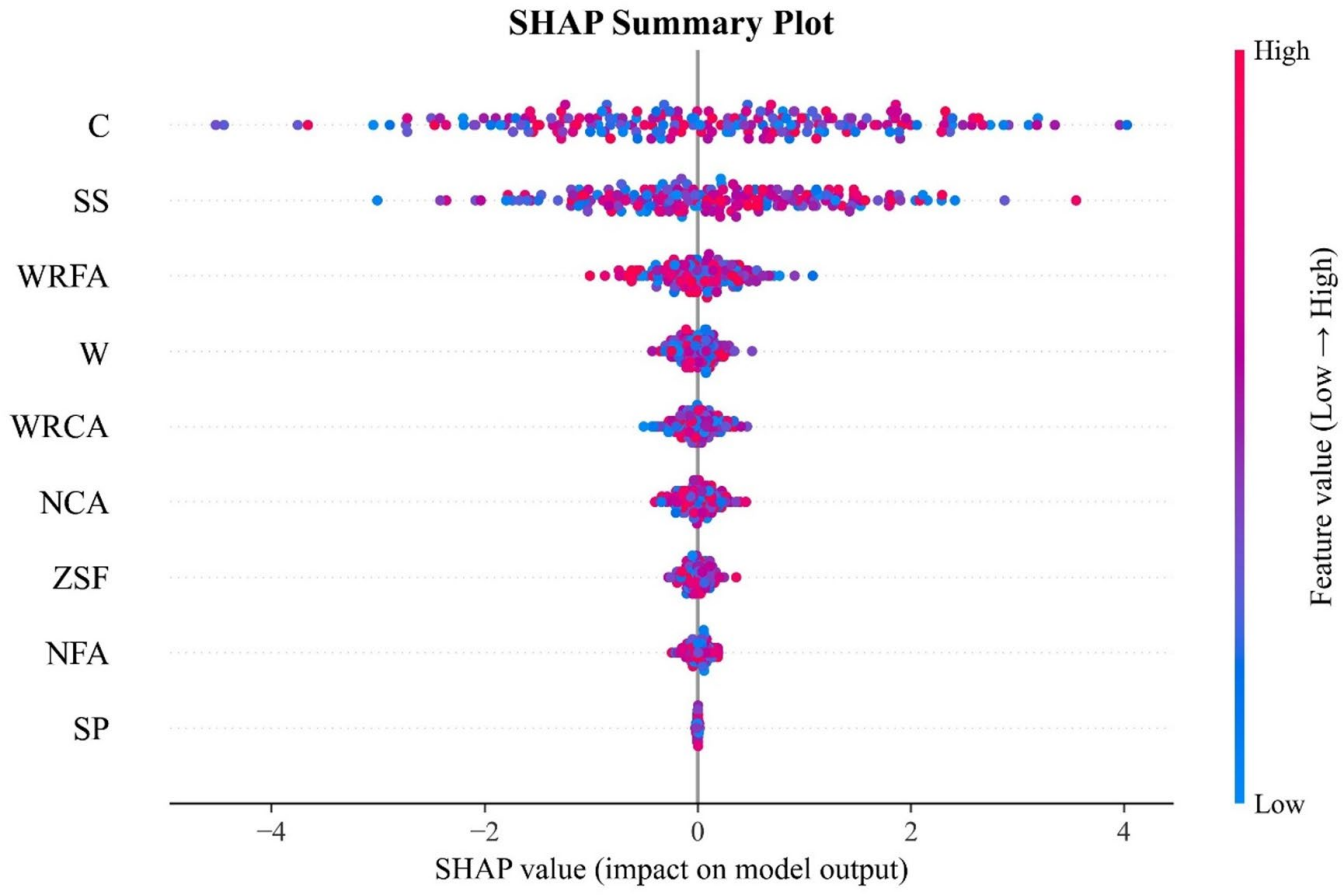
SHAP feature importance on compressive strength prediction.

**Fig. 17:**
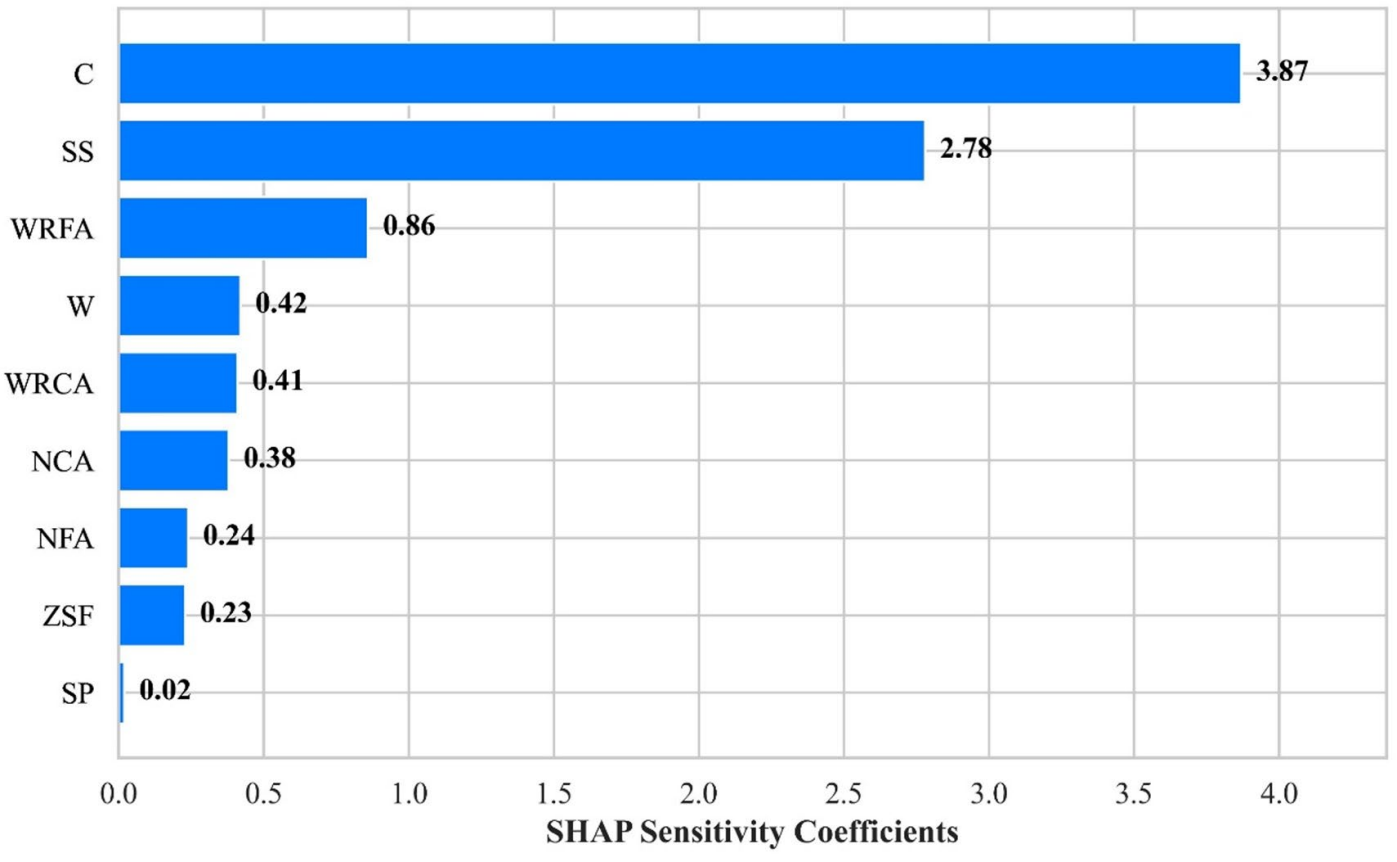
Mean SHAP values for relative contribution of input variables.

**Fig. 18:**
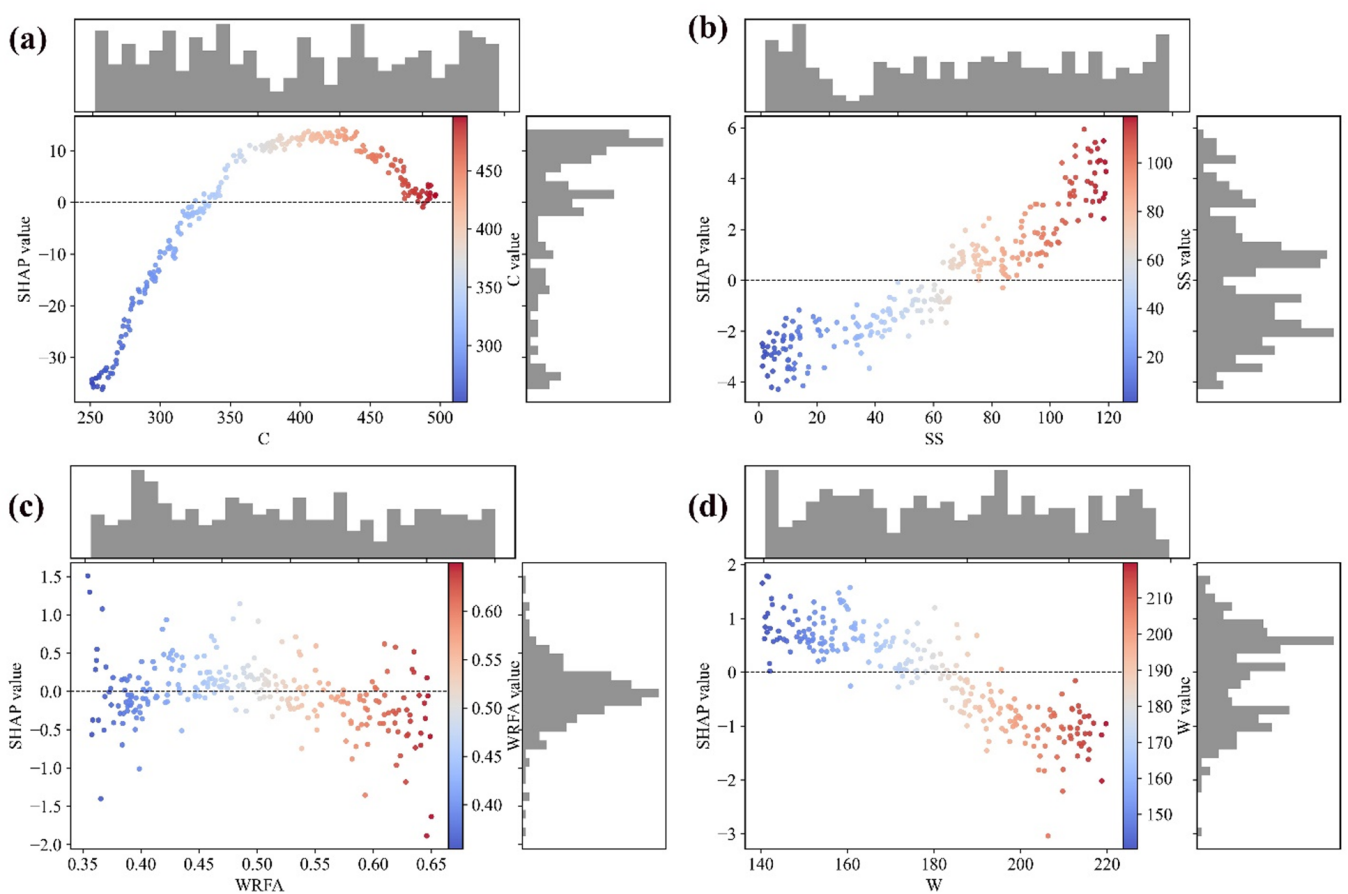
SHAP dependence plot of most influential variables (a) C, (b) SS, (c) WRFA, and (d) W.

Figure 16 presents the sensitivity analysis through SHAP summary plot for the optimized FPA-DNN model on compressive strength estimation. This reveals the relative contribution of each input parameter to compressive strength prediction along with the underlying physical mechanisms. It was identified that C exhibits the highest influence, as increased cement availability enhances hydration and promotes the formation of C–S–H gel, leading to strength improvement^82^. SS also shows a strong positive effect due to its latent hydraulic and pozzolanic activity. This can be prioritized during mix design to achieve target strength levels while minimizing material usage without compromising strength, thereby reducing cost and embodied carbon. WRFA demonstrates a moderate and mixed influence, reflecting the balance between improved particle packing at optimal replacement levels and potential strength reduction associated with increased porosity at higher contents. This indicate that WRFA replacement should be maintained within the optimal range upto 50% to be beneficiary for the utilization in rigid pavement construction. Other parameters, including W, WRCA, NCA, ZSF, NFA, and SP exhibit comparatively lower impacts within the adopted mix design limit which indicate their secondary role towards the strength development. Figure 17 depicts the mean SHAP values for relative contribution of input variables. This provides further evidence that C and SS are the dominant factors governing compressive strength as contribute first and second rank respectively, while WRFA contributes a secondary role for the improving the concrete strength. The remaining parameters exhibit comparatively lower influence, indicating their supportive contribution within the considered mix design domain for the construction of rigid pavements. Figure 18 illustrates the SHAP dependence plots for the most influential input variables, (a) C, (b) SS, (c) WRFA, and (d) W highlighting their nonlinear effects on compressive strength prediction. From this plot, it was concluded that C and SS are the primary contributors to compressive strength development, exhibiting strong positive nonlinear effects, while WRFA influences strength in a sensitive, proportion-dependent manner. Higher water content adversely affects strength due to matrix dilution and increased porosity, confirming the importance of optimized mix proportioning for WRFA-based concrete.

### Partial dependence plot analysis

**Fig. 19:**
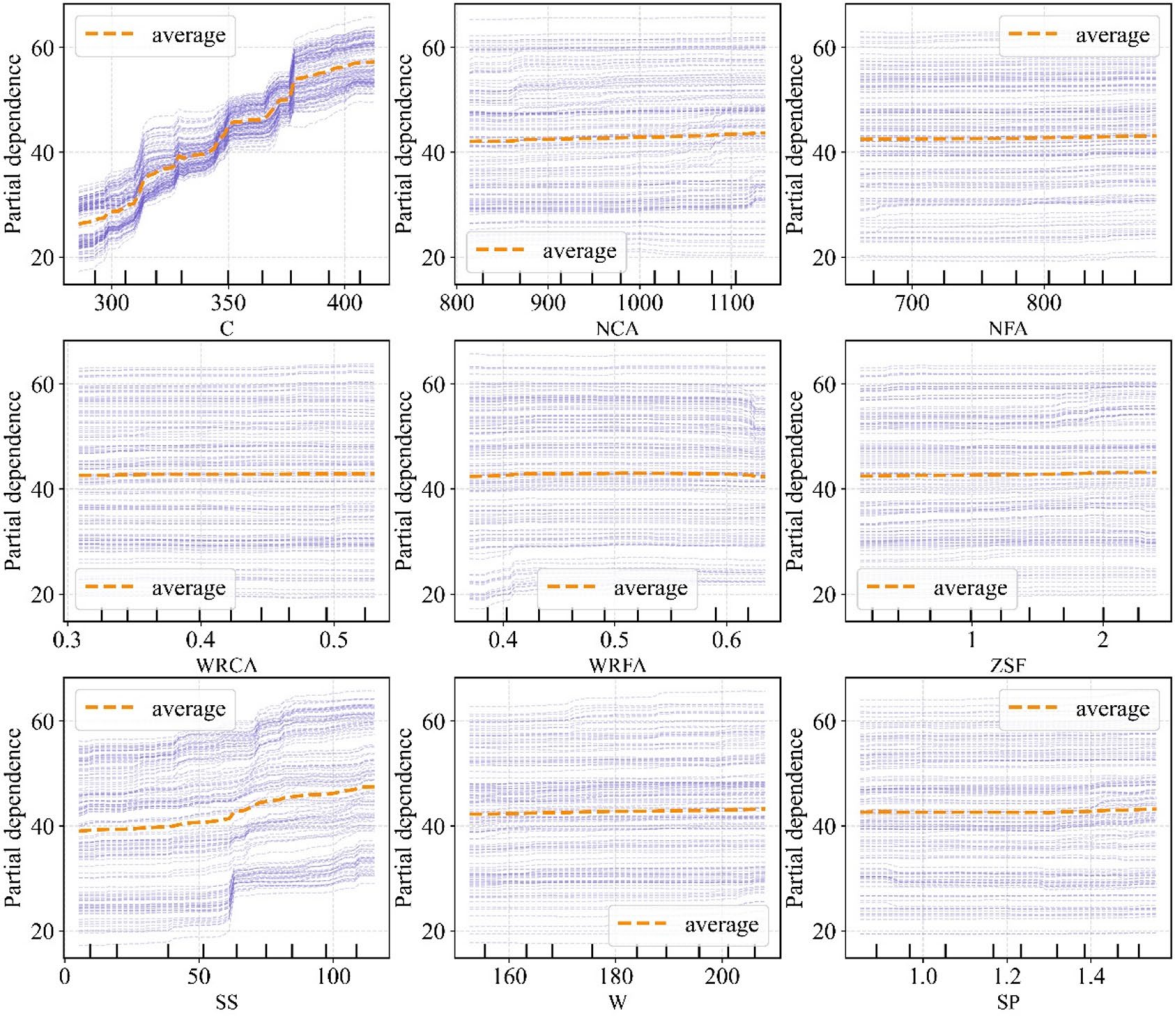
Visualization of non-linear interaction effects of input variables on compressive strength based on PDP- ICE curves.

Figure 19 presents PDP analysis with ICE curves for the input variables influencing compressive strength. The orange mark represents the average partial dependence, while the blue colour ICE curves illustrate individual sample-level responses. It was observed that C exhibits a strong positive and nonlinear relationship with compressive strength, confirming its dominant influence on strength development. The SS variable also shows a clear increasing trend indicating its beneficial contribution through secondary hydration and matrix densification. In contrast, parameters such as NCA, NFA, WRCA display relatively flat PDPs, suggesting a limited marginal effect on strength within the investigated ranges. Moreover, WRFA shows a mild nonlinear response, highlighting its sensitivity and the importance of optimized proportioning for enhancing strength performance. The remaining variables like ZSF and SP indicate modest positive contributions with limited variability.

### Development of graphical user interface

**Fig. 20:**
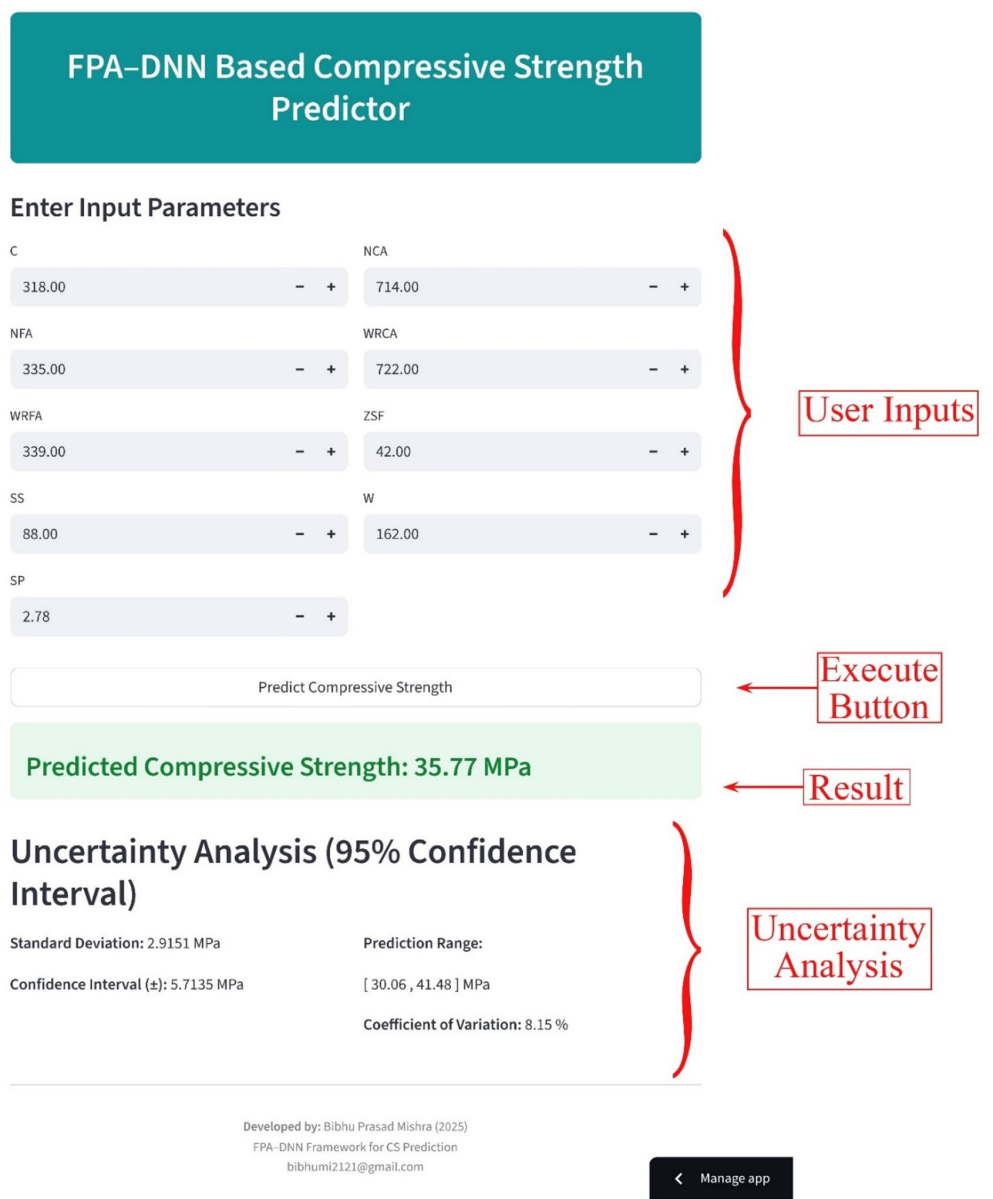
Development of GUI for compressive strength estimation.

An interactive GUI was developed to enhance the practical usability of the proposed predictive framework. As shown in Fig. 20, the GUI allows users to conveniently input the desired concrete mix parameters and instantly obtain the corresponding compressive strength prediction. All input parameters accepted by the GUI are explicitly defined in terms of units and permissible ranges, which correspond exactly to the minimum and maximum values observed in the training dataset. These ranges were established to ensure consistency with the experimental and literature-derived data used to develop the predictive models^83^. The interface incorporates the optimized FPA-DNN model as its backend, ensuring that the outputs remain consistent with the model’s validated performance. This user-friendly platform eliminates the need for programming expertise, making the tool accessible for engineers, researchers, and field practitioners seeking rapid and reliable strength. The developed GUI for compressive strength estimation 95% uncertainty analysis is publicly accessible at https://tinyurl.com/fpa-dnn.

### Sustainability assessment

To support the sustainability claims of the proposed SS20 + 50% WRFA concrete mix, an indicative cement-saving and embodied-carbon (CO_2_) reduction analysis was conducted. In the recommended mix, 20% of cement is replaced by SS, directly reducing the cement content required per cubic meter of concrete. Table 7 presents a simplified calculation illustrating the potential cement savings and associated reduction in embodied carbon for the SS–based concrete mix. It was found that a cement saving of 92 kg/m³ was achieved by replacing cement with steel slag, reducing the cement content from 460 kg/m³ in conventional concrete to 368 kg/m³ in the SS mix. As per the Intergovernmental Panel on Climate Change (IPCC) report, the average emission factor of 0.85 kg CO₂/kg of cement, this reduction translates to an estimated decrease of approximately 78 kg CO₂/m^3^ of concrete. Therefore, this assessment represents a first-order estimation intended to support the eco-efficiency claim for the rigid pavement construction.

**Table 7:** Calculation of cement Saving and CO₂ reduction for SS20 + 50% WRFA mix.

Sl. No	Description	Unit	Calculated Values
A	Cement content of conventional concrete	kg/m³	460
B	Cement content of SS mix	kg/m³	368
C	Cement saved (A − B)	kg/m³	92
D	Cement replacement level	%	20
E	CO₂ emission factor for cement	kg CO₂/kg cement	0.85
F	CO₂ emissions in conventional concrete (A × E)	kg CO₂/m^3^	391
G	CO₂ emissions in SS mix (B × E)	kg CO₂/m^3^	313
H	Net CO₂ reduction (F − G)	kg CO₂/m^3^	78

## Conclusions

This study systematically investigated the mechanical performance of concrete incorporating WRFA together with supplementary materials ZSF and SS followed by the development of an advanced predictive modelling framework using ML technique. Experimental results demonstrated that the inclusion of WRFA leads to a reduction in compressive strength, with decreases of 29.6%, 25.2%, and 23.9% at 7, 28, and 90 days, respectively, relative to mixes made with natural aggregates. Among the various combinations, SS20 emerged as the most effective mixture, providing the highest strength improvement and outperforming ZSF and hybrid combinations. A similar trend was observed in the flexural and tensile behaviour, where WRFA replacement generally reduced performance, but mixes incorporating 20% SS showed minimal deterioration—only about 3% reduction in flexural strength and 3.8% reduction in tensile strength at 28 days indicating that SS20 provides an optimal balance between performance and sustainability.

Based on the performance evaluation, the R² values for kNN (0.85), RF (0.86), ANN (0.89), DNN (0.91), and FPA-DNN (0.96) confirm the superior predictive accuracy of the FPA-DNN architecture. Error metrics including RMSE, MAPE, MBE, and MAD also indicate consistent improvement from shallow models to deeper architectures, with FPA-DNN achieving the lowest error levels (Testing RMSE = 2.87) and demonstrating the highest overall accuracy. The accuracy matrix further reinforced these findings, showing a clear trend where FPA-DNN outperformed all other models, followed by DNN, ANN, RF, and kNN model. A noise-robustness analysis was also conducted to evaluate the stability of these models under simulated White Gaussian Noise, providing insights into their behaviour under uncertain or field-like input conditions. The results indicated that kNN and RF were more sensitive to increasing noise levels, ANN and DNN showed moderate resilience, while FPA-DNN exhibited the strongest robustness, maintaining high predictive accuracy even at elevated noise intensities. This demonstrates that optimization-enhanced deep neural networks are better suited for real-world applications where data distortion or measurement uncertainty is common. SHAP-based interpretability analysis revealed that C and SS are the most influential parameters governing compressive strength, while WRFA exhibited a proportion-dependent effect, emphasizing the importance of optimized mix design. The integration of a GUI-based implementation enhances the practical applicability of the proposed framework by enabling real-time, user-friendly strength prediction within the validated input domain.

### Practical implications

The findings of this study demonstrate clear potential for implementation in real-world projects towards the economic and environmental benefits. The identification of an optimal mix proportion incorporating 50% WRFA and 20% SS offers a practical guideline for engineers to design sustainable concrete without compromising mechanical performance. The improved strength characteristics observed in the optimal mix indicate that recycled and industrial by-product materials can be effectively utilized in structural and pavement applications. From an operational perspective, the developed FPA–DNN model integrated with GUI, enables practitioners to predict compressive strength in real time based on input mix parameters. This reduces reliance on time-consuming laboratory trials and supports rapid decision-making during mix design, quality control, and material optimization processes. The inclusion of uncertainty bounds further enhances reliability by allowing engineers to account for prediction variability. Economically, the partial replacement of cement with SS leads to a measurable reduction in material cost, while the use of WRFA reduces expenses associated with natural sand procurement and waste disposal. Environmentally, the proposed mix design contributes to sustainability by lowering cement consumption—thereby reducing associated carbon emissions (CO₂) and by promoting the reuse of RAP and industrial waste materials. Additionally, reduced extraction of natural aggregates supports conservation of natural resources and minimizes environmental degradation.

## Limitations and future research directions

Despite the promising results obtained in this study, several limitations should be acknowledged. First, the dataset used for model development comprises 264 samples, which, although sufficient for reliable model training and validation, may not fully represent the variability associated with recycled fine aggregate concrete produced under different curing conditions, material sources, and environmental exposures. Expanding the dataset with large-scale experimental and field-based data would further enhance model robustness and generalizability. Second, the present study focuses primarily on compressive strength as the target output. Durability-related properties such as drying shrinkage, permeability, water absorption, sorptivity, chloride penetration, and resistance to chemical attack were not examined. Future research will extend the experimental program to include comprehensive durability testing, along with microstructural characterization, to establish the long-term serviceability of WRFA and SS–based concrete. Third, while the proposed FPA-DNN model demonstrated superior predictive accuracy, the framework relies on supervised learning and assumes high-quality input data. Uncertainty arising from experimental noise and data imbalance was not explicitly modeled. Future research may explore uncertainty-aware or probabilistic ML approaches to improve prediction reliability. Additionally, while SHAP-based interpretability provides valuable insights, further integration of physics-informed or hybrid modeling approaches could enhance model transparency and reliability. Finally, GUI was developed to enhance practical usability, real-world validation through industry-scale implementation remains necessary. Future studies may also integrate life cycle assessment (LCA), cost analysis, and carbon footprint indicators to support decision-making in sustainable construction practices, as suggested in recent studies.

**Link to the GUI**: https://tinyurl.com/fpa-dnn.

## Data Availability

All data used in this study is available with the authors and will be provided on request.

## Abbreviations

C: Cement
NCA: Natural coarse aggregate
NFA: Natural fine aggregate
RAP: Recycled asphalt aggregate
WRCA: Washed recycled coarse aggregate
WRFA: Washed recycled fine aggregate
ZSF: Zirconia silica fume
SS: Steel slag
W: Water
SP: Super-plasticizer
W/C: Water to cement ratio
SCM: Supplementary cementitious material
RCA: Recycled concrete aggregate
IRC: Indian road congress
IS: Indian standard
SEM: Scanning electron microscope
ITZ: Interfacial transition zone
ML: Machine learning
kNN: k-nearest neighbor
RF: Random forest
ANN: Artificial neural network
DNN: Deep neural network
FPA: Flower pollination algorithm
MAPE: Mean absolute percentage error
RMSE: Root mean square error
R^2^: Coefficient of determination
MBE: Mean bias error
MAD: Mean absolute deviation
CI: Confidence interval
XAI: Explainable Artificial Intelligence
SHAP: SHapley additive explanations
PDP: Partial dependence plot
ICE: Individual conditional expectation
AWGN: Additive white gaussian noise
GUI: Graphical user interface
